# Leveraging Dissolved Organic Matter Collections as a Natural Chemical Library to Link Molecular Traits with Cellular Morphological Responses

**DOI:** 10.1021/acs.est.5c12756

**Published:** 2026-03-02

**Authors:** Xin Zhang, Mourad Harir, Joel Schick, Marianna Lucio, E. Michael Perdue, Philippe Schmitt-Kopplin

**Affiliations:** † Research Unit Analytical Biogeochemistry, Helmholtz Munich, Neuherberg 85764, Germany; ‡ Chair of Analytical Food Chemistry, Technical University of Munich, Freising 85354, Germany; § Genetics and Cellular Engineering Group, Research Unit Signaling and Translation, Helmholtz Munich, Neuherberg 85764, Germany; ∥ School of Earth and Atmospheric Sciences, 1372 Georgia Institute of Technology, 311 Ferst Drive, Atlanta, Georgia 30332-0340, United States

**Keywords:** DOM, IHSS standards, FT-ICR MS, cell painting assay, environmental bioactivity

## Abstract

Dissolved organic matter (DOM) exhibits a highly complex molecular composition and maintains ecosystem stability, acting as a crucial interface between biotic and abiotic processes. Although DOM’s molecular complexity and biological effects are widely studied, most investigations use targeted bioassays, examining specific responses and linking molecular features only to predefined biological outcomes when assessing potential bioactive components. Here, we analyzed International Humic Substances Society (IHSS) reference standards of natural organic matter (NOM) and humic fractions, including humic acids (HAs) and fulvic acids (FAs), using ultrahigh resolution Fourier- transform ion cyclotron resonance mass spectrometry (FT-ICR MS) alongside the Cell Painting (CP) assay, a multiplexed, image-based morphological profiling method. The chemical composition of IHSS samples was influenced by fractionation methods and environmental sources. HAs exhibited stronger aliphatic and aromatic characteristics, whereas FAs and NOM extracted by reverse osmosis were more oxidized. Distinct molecular patterns were observed among terrestrial HAs, Pony Lake FAs, terrestrial FAs, and other fractions. In the CP assay, the most hydrophobic humic substances induced the most pronounced morphological changes. Linking chemical features with morphological outcomes suggested lipid-like compounds and nitrogen-rich aromatic species as likely contributors. This integrative approach provides preliminary molecular leads for further isolation, structural characterization, and mechanistic studies of DOM bioactivity.

## Graphical abstract

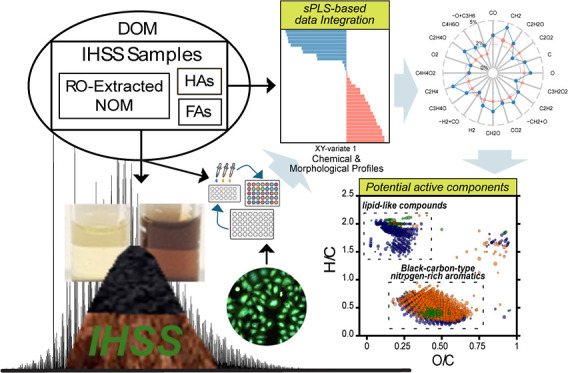

## 1 Introduction

Organic matter (OM) is one of the most abundant and reactive reservoirs in global element cycles, linking living and mineral components and present through all earth environments, from atmosphere, waters, soils to deep geosphere,^1^ and has been involved in the evolution of life since planetary formation.^2^ Its variable behavior across these compartments influences global carbon cycling and climate feedbacks, modulated by biological and geochemical or physicochemical stressors.^3–5^ Despite OM’s global importance in climate regulation and Earth’s homeostasis, its chemical composition, behavior toward biology and transformation remain partially understood.^6,7^ For practical purpose, OM is operationally categorized into dissolved organic matter (DOM) and particulate organic matter (POM) based on filtration methods rather than intrinsic chemical differences.^8^ Among them, DOM has attracted more attention due to its high mobility and reactivity,^9^ which enable it to play a central role in critical ecosystem services such as nutrient cycling, carbon sequestration, pollutant transport, soil fertility, microbial dynamics, and water quality.^10,11^ Differential fractions within DOM are responsible for key properties and processes, making their chemical characterization fundamental for understanding DOM’s complex composition and biogeochemical functions.^12,13^ To support research in this area, the International Humic Substances Society (IHSS) has developed a library of standards and reference materials, sourced from representative environments and extracted using consistent protocols to promote comparability and reproducibility in DOM studies.^14,15^

DOM originates from the combined effects of biotic and abiotic degradation and transformation of plant and microbial residues across diverse environments.^16–19^ Abiotic processes, such as photochemical and chemical oxidation, can occur widely in natural environments and play a major role in the formation and transformation of oxidized natural organic matter (NOM), alongside biological processes. These diverse environmental origins and varying degrees of decomposition result in a complex and heterogeneous composition of DOM, which limits its direct characterization.^20^ Moreover, DOM occurs at low concentrations in natural systems, making extraction and concentration essential for molecular-level analysis.^21^ In aquatic settings, reverse osmosis (RO) techniques have enabled the extraction of natural organic matter (NOM) under mild conditions, typically recovering 80–90% of the total organic carbon.^6^ The RO-extracted materials reflect the functional and structural complexity of DOM and are critical for investigating its ecological behavior.^22^ Also, solid-phase extraction (SPE) using styrene-divinylbenzene polymer (PPL) cartridges has become widely used in recent years due to its simplicity, efficiency, and suitability for field extractions in a broad range of DOM.^23^ To ensure consistency and comparability, IHSS has established standard alkaline extraction protocols for diverse materials. Following these protocols, DOM can be fractionated in standard operation procedures based on solubility in acids and bases into (i) humic acids (HAs), which precipitate at pH lower 2, (ii) fulvic acids (FAs), which remain soluble at all pH levels and (iii) humins, the highly molecular insoluble and hydrophobic organo-mineral fraction. These operational categories effectively represent the nature of DOM, facilitating the study of DOM’s diverse reactivity and environmental functions over decades.^24^

The complex and heterogeneous composition of DOM fractions also present a significant challenge for analytical techniques. Although conventional chemical analyses yield bulk compositional data, they often do not capture the molecular features necessary to connect structure with ecological or biological function. Fourier-transform ion cyclotron resonance mass spectrometry (FT-ICR MS) addresses this limitation by offering unparalleled mass accuracy, resolution, and dynamic range.^25^ It enables nontargeted molecular characterization of thousands of compounds from minimal sample quantities, providing unprecedented insights into DOM composition, reactivity, and transformation pathways.^26^ Such efforts are crucial for identifying consistent patterns in DOM behavior, for establishing molecular indicators of ecosystem processes, and for refining predictive models of OM dynamics under global change. Although FT-ICR MS has been widely applied to the chemical characterization of DOM fractions, comparative studies of DOM fractions from different sources remain limited to case studies, such as single-watershed comparisons, pre- and post-disturbance analyses, depth-resolved soil or peat profiles, or evaluations of extraction-method effects within the same environment.

Meanwhile, DOM fractions are attracting growing attention for their bioactivity.^27,28^ This interest arises from both the need to understand their ecological functions and from their growing use in humic-based products for green agriculture, as well as in health-related and cosmetic applications.^29,30^ While DOM’s biogeochemical relevance is well recognized, its potential in biological contexts remains underexplored. Recent studies have revealed that its complex molecular composition can exhibit diverse biological activities, including antiviral, anti-inflammatory, and antibacterial effects, highlighting its potential for medicinal and health-related applications.^31–33^ The polyfunctional and highly complex mixture nature of DOM, however, presents challenges for characterizing its biological effects. Current studies often rely on target-based assays, which may miss off-target effects or provide limited insight into mechanisms of action (MOA).^34^ To overcome these limitations, phenotypic assays have emerged as powerful alternatives. Rather than focusing on predefined targets, they assess the overall effects of treatments on biological systems, allowing target-agnostic activity profiling.^35^ Among these, the Cell Painting (CP) stands out for its high-throughput capability, cost-effectiveness, and ability to generate rich morphological data.^36^ By applying fluorescent dyes to visualize multiple cellular structures, CP provides a nontargeted readout of cellular responses and can detect polypharmacological effects.^37,38^ It has been successfully applied to assess the bioactivity of various chemicals, gene perturbations, and natural products,^39–41^ making it particularly suited for the complex and multifunctional nature of DOM.

In this study, FT-ICR MS and CP were integrated to perform detailed chemical and morphological profiling of 33 diverse IHSS standard and reference samples, which represent a broad range of DOM fractions from diverse environments. While traditional target-based assays are limited in capturing the multidimensional cellular responses to complex DOM mixtures, our integrated CP-FT-ICR MS approach enables high-content morphological profiling directly linked to molecular composition, providing a more comprehensive understanding of potential bioactive components. This allowed for a systematic evaluation of how origins and extraction methods influence the molecular composition and morphological effects of DOM. More importantly, this study presents a statistical framework for identifying potential bioactive components within DOM fractions by linking chemical profiling with multiparametric bioactivity assay results. Overall, this study bridges the molecular and biological complexity of DOM, offering a novel approach to understanding its functional roles in both environmental and health-related contexts.

## 2 Materials and Methods

### 2.1 Sample Description

All samples used in this study were obtained from the IHSS, with source material descriptions available on its homepage (https://humic-substances.org). As summarized in Table S1 and Figure S1, standard materials were collected from the Elliott Series, Pahokee Series, Bowman County (Leonardite), and the Suwannee River, in accordance with the strict IHSS criteria. Reference materials from other sources were prepared following slightly less stringent standards. All samples were dissolved in dimethyl sulfoxide (DMSO) to prepare 10,000 ppm stock solutions. The solutions were thoroughly vortexed, sonicated, and centrifuged, and the resulting stock solutions were stored at −20 °C for subsequent experiments.

### 2.2 Molecular Characterization of IHSS Samples

The molecular compositions of IHSS samples were analyzed using a high-field Fourier-transform ion cyclotron resonance mass spectrometer equipped with electrospray ionization (ESI) operated in negative ion mode. Thousands of signals were translated into elemental formulas and visualized using van Krevelen-type diagrams (H/C vs O/C) and related plots (H/C vs *m*/*z*), with each formula represented as a dot. Dot size indicates relative abundance, providing a visual overview of oxygenation (O/C) and saturation (H/C) across molecular classes (CHO, CHNO, CHOS, and CHNOS). Detailed instrumental parameters, calibration procedures, and data processing workflows are provided in Text S1.

### 2.3 Cell Line and Cell Culture

U2OS cells were cultured in Dulbecco’s Modified Eagle Medium (DMEM; Gibco, cat. 31885-023) supplemented with 10% fetal bovine serum (Gibco, cat. 10270-106), 1% Penicillin–Streptomycin (Gibco, cat. 15140-122), and 1% nonessential amino acids (NEAA; Gibco, cat. 11140-035). Cells were maintained in the incubator of 95% humidity and 5% CO_2_ atmosphere and were cultured up to passage 8.

### 2.4 Cell Painting

The CP was performed following the originally published protocol.^37^ U2OS cells were seeded into a Poly-D-Lysine -coated 384-well plate (PerkinElmer, cat-781091) at a density of ∼1000 cells per 50 μL per well and incubated under standard culture conditions for 24 h. Cells were then exposed to various samples. Briefly, stock solutions of each sample were diluted 100-fold in culture medium and used to replace the existing medium in each well, ensuring a final DMSO concentration of 1% across all treatments. Each sample was measured in six replicates to ensure reproducibility.

After 24 h of incubation, live cells were first labeled with Mito Tracker^TM^ Deep Red, followed by fixation with 4% paraformaldehyde (PFA) and permeabilization. Cells were then stained with five additional fluorescent dyes for 30 min. In total, eight cellular components were labeled in this assay. Detailed information on the fluorescent dyes is provided in Table S2.

Fluorescent images were collected from five channels using the CellInsight^TM^ CX7 high-content analysis platform (Thermo Fisher Scientific), equipped with a 20× objective (Olympus^TM^, 0.4 NA). Image analysis was conducted using Compartmental Analysis BioApplication and Morphology Explorer (Cellomics, Thermo Fisher Scientific) to extract morphological features from individual cells. These features describe the number, fluorescence intensity, area, and shape of different stained cellular compartments, and collectively constitute a quantitative representation of cellular morphology. Well-level morphological features were then generated by aggregating these single-cell measurements. For each well, the mean of each single-cell feature was calculated, and additional descriptors including the variance and the correlations among these mean values were calculated to capture population-level changes in morphology. In total, 272 well-level morphological features were obtained and subsequently used for multivariate analysis and data integration analysis in this study. Detailed information about the name, analysis modules, imaging channels, and feature categories of these features are provided in Table S3. Cell-level features were retained separately for evaluating single-cell distributions. For both well-level and cell-level measurements, feature values were normalized to the corresponding vehicle controls (cells treated with 1% DMSO on the same measurement plate) using the robust MAD (rMAD) method to reduce technical variability.^37,42^ Specifically, the median of the feature values from the vehicle controls was subtracted from each treatment value, and the result was then divided by the median absolute deviation (MAD) of the control group. The normalized morphological features were subsequently expressed as z-scores and used for downstream analysis.

To assess the autofluorescence of samples and their potential interactions with CP dyes, a parallel experiment was conducted under CP conditions but without cells. Fluorescence was measured for individual samples, dyes, and their mixtures at four concentrations (1, 10, 50, and 100 ppm). Readouts were obtained using an EnVision 2104 Multilabel Plate Reader (PerkinElmer), applying the same wavelengths used in the CP. Sample-derived autofluorescence was evaluated by subtracting the fluorescence of each sample to that of phosphate-buffered saline (PBS). Differences in fluorescence between dye-sample mixtures and dyes alone were used to evaluate sample-induced effects on dye fluorescence.

### 2.5 Statistical Analyses and Data Visualization

All data processing, statistical analyses, and data visualization were carried out using R (version 4.4.1) and Python (version 3.9.13). Hierarchical clustering analysis (HCA) was performed with the Ward.D2 method and Euclidean distance using the ComplexHeatmap package (version 2.20.0). Principal component analysis (PCA) was conducted with FactoMineR (version 2.6) and Factoextra (version 1.0.7).

Sparse Partial Least Square (sPLS) was performed using mixOmics (version 6.28.0) in regression mode to explore the association between the FT-ICR MS and CP data sets. For data preprocessing, CP data were summarized by calculating the median well-level morphological profiles of six replicates for each sample. FT-ICR MS peak intensities were first normalized to the total intensity of each sample, then scaled to unit variance across samples for each peak. The resulting FT-ICR MS and CP data sets were merged and subjected to z-score normalization by sample. The sPLS model was optimized by selecting the number of components that maximized the Q2.total through 10-fold cross-validation repeated 5 times. A Q2.total threshold of 0.0975 was used as a cutoff to determine the number of components retained in the model. Components with Q^2^.total values above this threshold were considered to provide nonrandom predictive contribution under cross-validation, whereas components below this threshold were excluded from interpretation.^43^ Feature selection was performed using lasso penalization on the loading vectors, selecting the subset of variables that maximized the cross-validated correlation between the two data sets. Among the selected FT-ICR MS features, a subset associated with strong morphological effects was further analyzed using mass network analysis in Gephi (version 0.10.1). This network was constructed based on exact mass differences to reveal potential molecular connections and transformation pathways.

## 3 Results

### 3.1 Profiling the Molecular Composition of IHSS Samples

Comprehensive molecular profiling of 33 IHSS samples was conducted using FT-ICR MS, with materials collected from diverse environments including soil, peat, lignite (coal), and inland freshwater. The samples were categorized into terrestrial (HAs and FAs) and aquatic (HAs, FAs, and NOM) groups, enabling an in-depth assessment of how environmental origins and extraction protocols influence the molecular composition of DOM fractions (Table S1). A unified solvent system, dissolving samples in DMSO and diluting with methanol, was used to facilitate consistent comparisons across all DOM fractions. This approach effectively addressed solubility challenges, particularly the limited resolubility of HAs postextraction.^44^ The molecular compositions were grouped by the number of oxygen (O), nitrogen (N), and sulfur (S) atoms to explore heteroatom compositions, revealing the molecular diversity across environments. The relative abundance of these heteroatom classes varied across samples (Figure S2), with terrestrial HAs and Pony Lake FA (PLFA) containing fewer oxygen atoms, indicating lower oxidation. Elevated levels of N- and S-containing compounds were observed in terrestrial HAs, terrestrial FAs, and PLFA, suggesting that environmental factors dominate the variability in DOM’s N- and S-content. The chemical composition of the IHSS samples was subsequently examined by classifying assigned formulas based on their elemental composition and predefined compound classes (Figure 1a). The elemental composition demonstrated a strong source dependence, with terrestrially derived samples exhibiting a notable presence of N-containing constituents in all fractions, while aquatic samples were characterized by CHO formulas, except for PLFA, which was enriched in N- and S-containing compounds. Compound class distribution was influenced by both environmental sources and fractionation schemes. Highly unsaturated and phenolic compounds dominated NOM, FAs, and aquatic HAs, whereas soil HAs were enriched in aromatic and aliphatic compounds, and PLFA showed a relatively higher proportion of aliphatic compounds.

**Figure 1:**
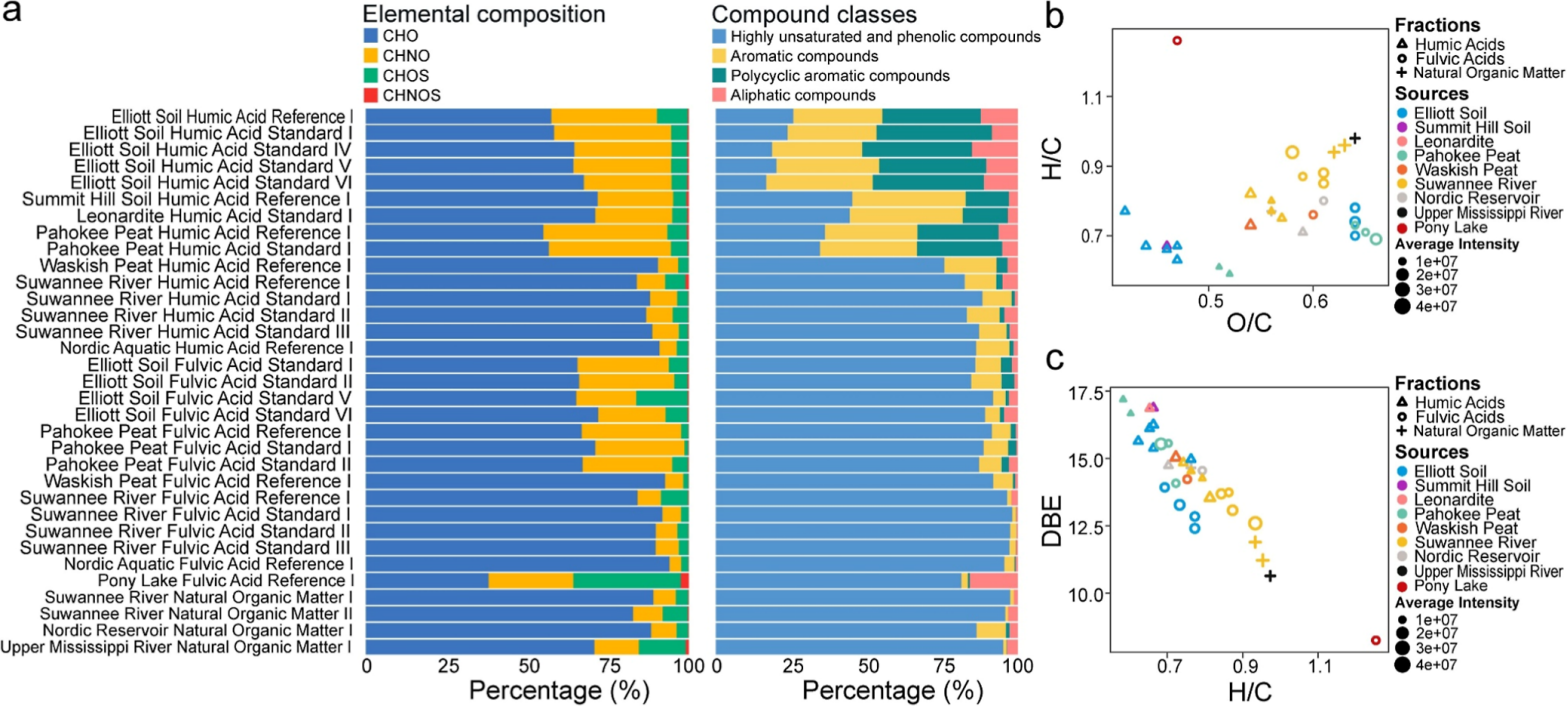
Molecular characterization of International Humic Substances Society (IHSS) samples revealed by Fourier-Transform Ion Cyclotron Resonance Mass Spectrometry (FT-ICR MS). (a) Stacked bar plots showing the proportions of assigned formulas categorized by elemental composition (left panel: CHO, CHNO, CHOS, CHNOS) and by compound class (right panel), including highly unsaturated and phenolic compounds (AI_mod_ ≤ 0.5 and H/C < 1.5), aromatic compounds (0.5 < AI_mod_ ≤ 0.67), polycyclic aromatic compounds (AI_mod_ > 0.67), and aliphatic compounds (AI_mod_ ≤ 0.5 and H/C ≥ 1.5).^51^ (b) Intensity-weighted average hydrogen-to-carbon (H/C) versus oxygen-to-carbon (O/C) ratios for each sample. (c) Intensity-weighted double bond equivalent (DBE) versus H/C ratios for each sample.

Van Krevelen diagrams (Figure 1b) provided further insights into molecular compositions, with terrestrial HAs exhibiting a strong presence of aromatic and condensed compounds, reflected by low hydrogen-to-carbon (H/C) and oxygen-to-carbon (O/C) ratios, characteristic of their stable, aromatic nature. In contrast, aquatic fractions, Waskish Peat HA and Waskish Peat FA (WPHA and WPFA), and terrestrial FAs were more oxygenated (oxidized) and aliphatic, showing higher H/C and O/C ratios. Among these, terrestrial FAs had the highest oxygenation state, which may reflect the introduction of oxygen-rich functional groups during microbial alteration and partial photo-oxidation.^45,46^ The comparison of average double bond equivalents (DBE) against H/C ratios (Figure 1c) showed a clear shift from aromatic terrestrial HAs to more aliphatic aquatic FAs, reflecting differences in environmental sources and fractionation methods. WPHA and WPFA aligned more closely with aquatic materials, consistent with their origin in nutrient-poor, cold bog peat,^47^ in contrast to the agricultural peat environment of Pahokee Peat.^48^ These patterns support previous studies,^49,50^ confirming the aromatic nature of HAs and the oxygen-rich character of FAs and NOM.

### 3.2 Multivariate Analysis for Identifying Molecular Patterns of IHSS Samples

Hierarchical clustering analysis (HCA) was applied to assess the compositional patterns of IHSS samples based on their assigned molecular compositions. The resulting dendrogram (Figure S3a) identified four clusters. Cluster 1 contained all terrestrial HAs except WPHA. Cluster 2 consisted exclusively of PLFA. Cluster 3 grouped all terrestrial FAs except WPFA. Cluster 4 included all aquatic fractions together with WPHA and WPFA. A detailed list of the samples within each cluster is provided in Table S4. To reinforce these observations, we next performed principal component analysis (PCA). The PCA score plots (Figure S3b) showed strong agreement with the HCA results, providing consistent evidence for the sample clusters identified in HCA. Principal component 1 (PC1) effectively separated terrestrial HAs from other sample types, while PC2 further distinguished terrestrial FAs from aquatic fractions as well as WPHA and WPFA. PC3 highlighted the unique molecular signature of PLFA. Interestingly, the variance along PC1 and PC2 indicated partial molecular similarities between PLFA and terrestrial FAs. Since HCA and PCA revealed consistent cluster patterns, PCA loadings were used to identify the molecular features driving these clusters. Samples were divided into four groups based on their positions in the PCA score space, and for each group, molecular formulas with loading directions corresponding to the quadrant of the group were extracted. For instance, formulas with negative PC1 loadings were selected for terrestrial HAs in the negative PC1 region, and formulas with positive loadings on both PC1 and PC3 were selected for PLFA in the quadrant with positive PC1 and PC3 scores. These group-specific molecular signatures were then visualized using van Krevelen and mass-edited H/C ratio diagrams (Figure 2), revealing distinct compositional patterns that contribute to the observed clustering.

**Figure 2:**
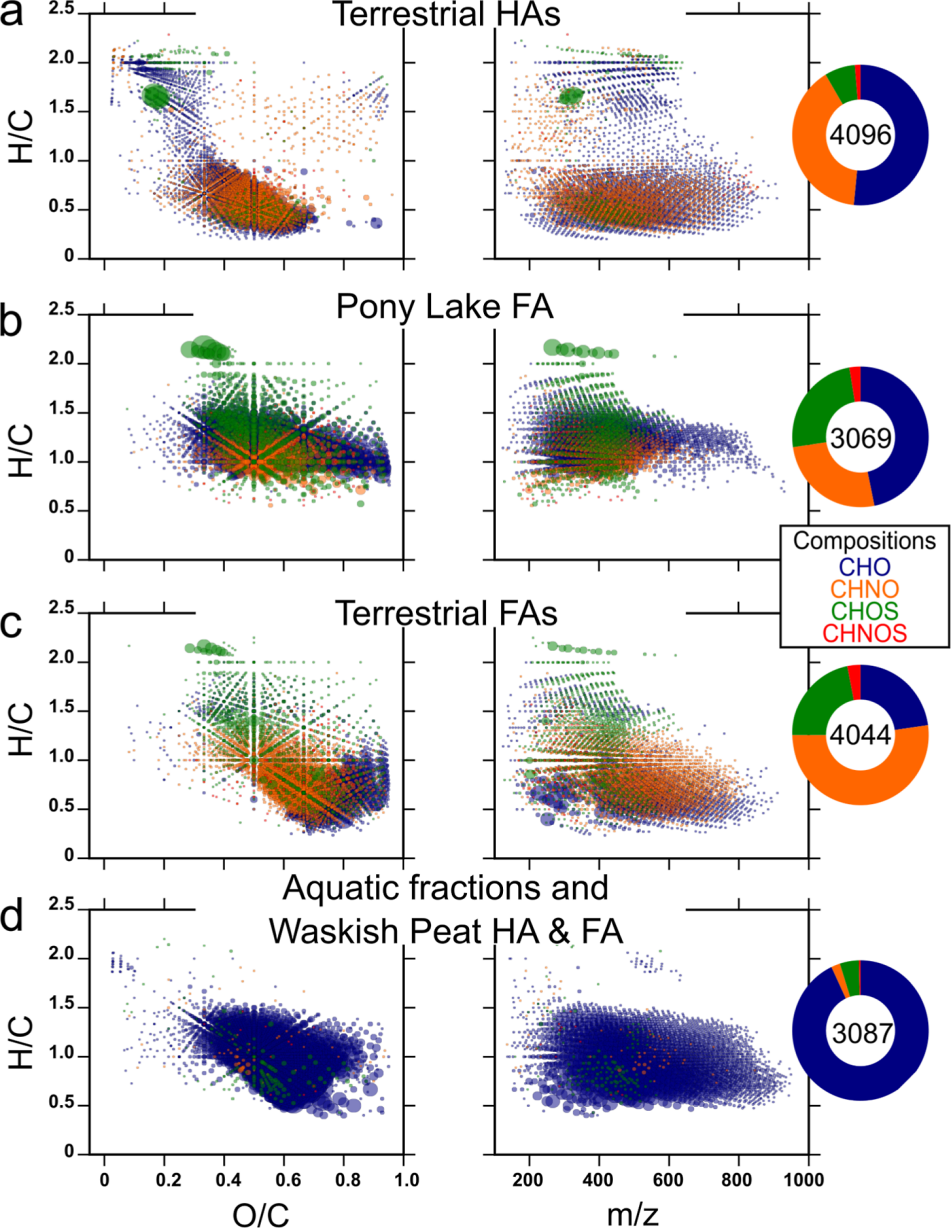
Van Krevelen diagrams showing hydrogen-to-carbon versus oxygen-to-carbon ratios, alongside mass-edited hydrogen-to-carbon ratio plots displaying hydrogen-to-carbon ratios against the molecular weight of compositions contributing to sample separation in the Principal Component Analysis: (a) Terrestrial humic acids (HAs), (b) Pony Lake fulvic acid (FA), (c) Terrestrial fulvic acids except Waskish Peat fulvic acid (FAs), and (d) Aquatic fractions, Waskish Peat humic acid and Waskish Peat fulvic acid (Waskish Peat HA & FA). Molecular classes are color-coded as follows: CHO (blue), CHNO (orange), CHOS (green), and CHNOS (red). Bubble sizes are proportional to mass peak intensity. The general classification of major molecular classes in the van Krevelen diagrams is provided in Figure S4, showing typical regions for fatty acids, carbohydrates, lignins, tannins, and condensed aromatics based on their H/C and O/C ratios.

Based on these PCA-derived molecular signatures, compositional differences emerged among the sample clusters. Terrestrial HAs, terrestrial FAs, and PLFA showed higher levels of N- and/or S-containing compounds compared to aquatic samples (Figure 2a–c), consistent with previous studies on terrestrial organic matter.^52–54^ Terrestrial HAs were characterized by aliphatic compounds, lignin-like compounds, and black carbon (BC)-type condensed aromatics (Figure 2a), typical of humic substances observed in soils.^55^ In contrast, PLFA exhibited a higher abundance of reduced molecular compositions, with molecular weights primarily below *m*/*z* 600 (Figure 2b). Terrestrial FAs, aquatic fractions, WPHA and WPFA were enriched in oxygen-rich, tannin-like compounds (Figure 2c,d). Within these samples, terrestrial FAs exhibited a markedly higher proportion of highly oxygenated tannins and N- and S-containing molecules, suggesting microbial transformations,^9^ whereas aquatic samples contained these compounds at lower relative abundance (Figure S5).

### 3.3 Cell Painting for Morphological Profiling of IHSS Samples

The CP assay was employed to evaluate the morphological effects of IHSS samples. Given the complexity of these samples, we first assessed their autofluorescence and potential interactions between the samples and fluorescent dyes to evaluate the reliability of morphological readouts. As shown in Figure S6a, varying levels of autofluorescence were observed, which are consistent with previous studies.^56^ HAs demonstrated stronger fluorescence quenching than FAs and NOM across multiple dyes and concentrations (Figure S6b), agreeing with earlier findings.^57–59^ The quenching effect was more pronounced at lower concentrations, likely due to the colloidal behavior of humic substances, where aggregation at higher concentrations limits their interaction with dyes, while dispersion at lower concentrations enhances it.^60^

Based on this evaluation, CP was performed on all IHSS samples at a final concentration of 100 ppm. The workflow of the CP assay is illustrated in Figure S7. Fluorescent images were visually examined to ensure accurate identification of cellular compartments, and morphological features strongly influenced by fluorescence intensity were excluded from further analysis. The resulting well-level morphological profiles were analyzed using PCA, revealing a progressive separation from vehicle controls, with samples ordered from NOM to FAs to HAs and from aquatic to terrestrial sources (Figure 3a), indicating increasing morphological activity. Consistently, the Global Euclidean Distance metric, which quantifies deviations from controls, differentiated IHSS samples into those with high CP activity (more morphologically active) and those with low CP activity (less morphologically active), revealing that terrestrial HAs exhibited greater morphological activity (Figure S8). This pattern is consistent with a recent study of HAs exhibiting greater antiviral activity than FAs,^61^ challenging the conventional assumption that FAs are more reactive due to their higher oxygen content.^62^ Notably, despite its high heteroatom content, PLFA did not show significant differences in morphological effects compared to other aquatic FAs. Further HCA of the median well-level morphological profiles revealed a consistent reduction in nuclear area and diameter in samples with higher CP activity (Figure S9). Single-cell distribution analysis supported these findings, showing minimal changes for treatments with low CP activity and a clear shift toward smaller nuclear areas and diameters in treatments with high CP activity, which are consistent with apoptosis-like or genotoxic responses, as reductions in nuclear size often reflect chromatin condensation and early cytotoxic stress (Figure 3b). Representative fluorescent images demonstrated nuclear shrinkage in response to terrestrial HA exposure (Figure S10). Although cell shrinkage was observed in different treatments, it was not initially recognized due to its common occurrence (see cell area panel in Figure 3b). Both nuclear and cell shrinkage are morphological hallmarks of apoptosis, and are often linked to DNA damage.^63^ The results suggest that terrestrial HAs may possess genotoxic potential, supporting previous studies that link HAs to apoptosis.^64–66^

**Figure 3:**
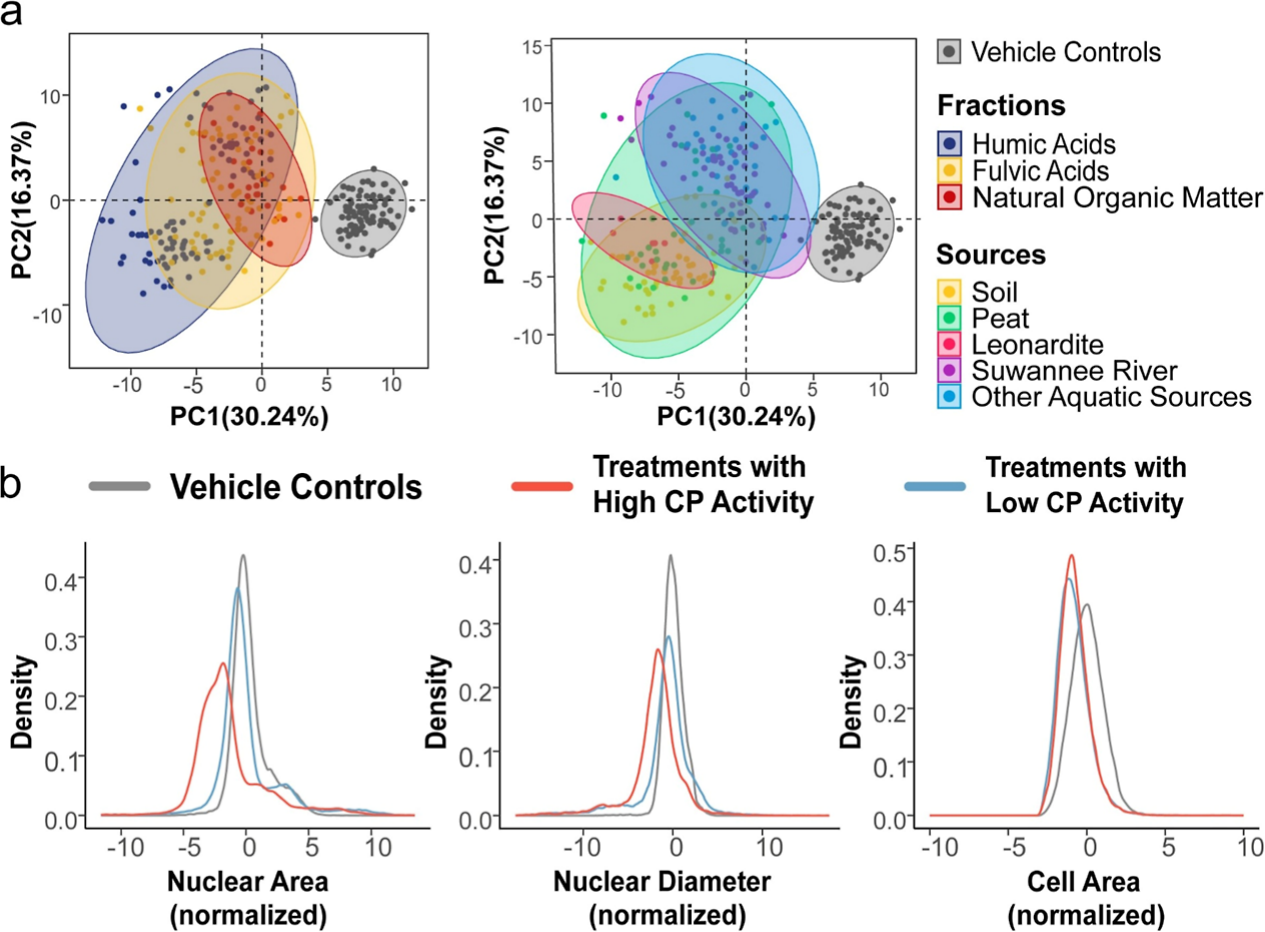
Morphological profiling of IHSS samples using the Cell Painting (CP). (a) Principal component analysis of well-level morphological profiles from U2OS cells exposed to IHSS samples. Both panels display the same score plot, colored by fraction types (left) and source materials (right). Vehicle controls (cells treated with 1% DMSO) are shown in gray. (b) Single-cell distributions showing differences in nuclear area, nuclear diameter, and cell area among controls, high-activity, and low-activity samples. For both well-level and cell-level measurements, morphological features were centered by subtracting the median of the control population and scaled by dividing by the corresponding median absolute deviation (MAD).

### 3.4 Integrating Chemical and Morphological Profiles to Identify Active Components in IHSS Samples

We employed a multivariate integration approach to identify the morphologically active components in IHSS samples by linking their chemical and morphological profiles. Sparse partial least-squares (sPLS) was selected for its ability to highlight the causal relationship between predictor variables and response variables.^43^ Model performance was assessed using the cross-validated Q2.total criterion based on repeated 10-fold cross-validation (5 repeats). A Q2.total threshold of 0.0975 was used to determine the number of components to retain.^43^ Only the first component exceeded this cutoff (Q2.total = 0.1333), whereas all subsequent components fell below the threshold. Therefore, the sPLS model was fitted using only the first component. Feature selection retained 2800 FT-ICR MS chemical features (X) and 80 CP morphological profiles (Y). On the first component, the fitted sPLS model explained 22% of the variance in the chemical features (X) and 33% of the variance in the morphological profiles (Y). Samples were projected onto the latent space defined by the integrated chemical and morphological profiles (Figure 4a). The first latent component (XY-variate 1) effectively distinguished terrestrial humic acids (HAs) from other IHSS fractions.

**Figure 4:**
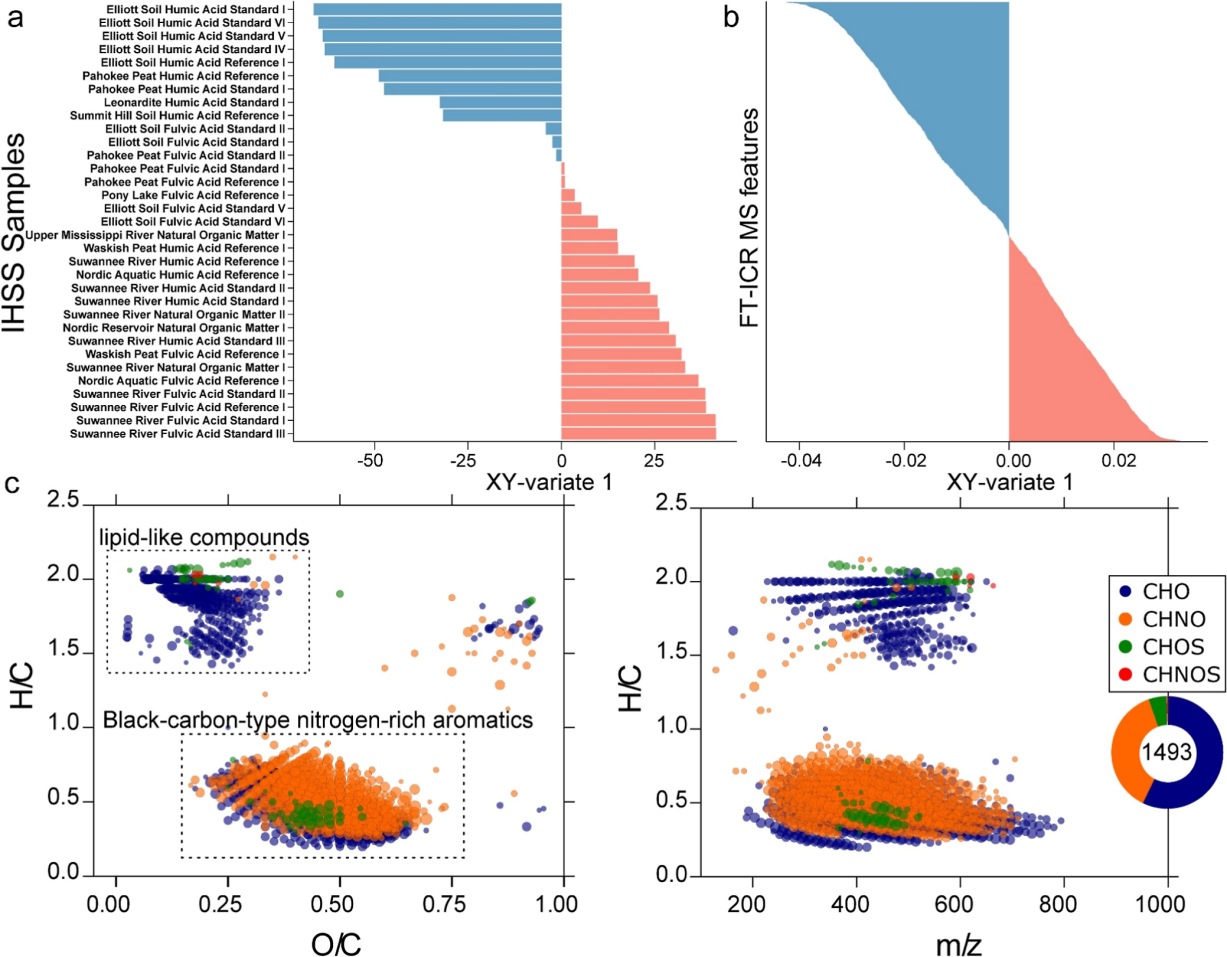
Integration of chemical and morphological profiles to identify morphologically active components in IHSS samples. (a) Projection of IHSS samples onto the first latent component (XY-variate 1) defined by integrated chemical (X: FT-ICR MS) and morphological (Y: Cell Painting) profiles. Samples projecting onto the negative axis of XY-variate 1 are labeled in blue, whereas samples projecting onto the positive axis are labeled in red. (b) Projection of FT-ICR MS chemical features (X) selected by the sPLS model onto XY-variate 1. Chemical features with negative loadings on XY-variate 1 are colored in blue, while those with positive loadings are colored in red, illustrating their contributions to the observed sample separation. (c) Van Krevelen (H/C vs O/C) and mass-edited H/C vs *m*/*z* diagrams depicting the chemical space of sPLS-selected features projected onto the negative axis of XY-variate 1. Two distinct compositional regions are observed, corresponding to lipid-like compounds and black-carbon-type nitrogen-rich aromatic compounds. Molecular classes are color-coded as CHO (blue), CHNO (orange), CHOS (green), and CHNOS (red), with bubble sizes proportional to mass spectral intensity.

FT-ICR MS chemical features selected by the sPLS model were projected onto XY-variate 1 to visualize their contributions to the observed sample separation (Figure 4b). We focused on the chemical features projected onto the negative axis of XY-variate 1 because these features were enriched in samples projecting in the same region of the latent space, corresponding to terrestrial humic acids, and were associated with morphological changes. The sPLS-selected molecular compositions projected onto the negative axis of XY-variate 1 clustered into two distinct regions on the van Krevelen diagram, corresponding to lipid-like and BC-type condensed aromatic compositions (Figure 4c). These molecular compositions exhibited moderate molecular weights and were enriched in N-containing species. To further explore the characteristics of these molecular compositions, we applied mass difference network analysis and Kendrick mass defect (KMD) analysis. The mass difference network revealed relationships based on exact mass differences, offering insights into transformation pathways.^67^ Meanwhile, KMD analysis detects homologous series differing by defined chemical functional groups and facilitates the identification of compound classes based on their repeating units. A mass difference network was constructed by matching the exact mass differences among all selected molecules with a predefined list of 40 mass differences corresponding to common functional groups and molecular transformations. Table S5 lists these transformations, showing each formula change and its chemical interpretation. The table includes common modifications observed in natural organic matter, such as alkylation, carbon addition, acylation, carbonylation, oxidation, hydroxylation, carboxylation, methylation, amino-related transformations, and diazomethylation, capturing both core structural modifications and functional group changes that drive molecular diversity and reactivity patterns. Analysis of the network (Figure S11a) revealed that the 20 most frequent mass differences were dominated by CH_2_–derived alkyl units (CH_2_, C_2_H_4_) and redox-related oxygen modifications (CO, O) (Figure S11b), indicating that alkylation and redox processes are the primary drivers of molecular transformations within both subnetworks. KMD analysis further revealed a strong presence of KMD (CH_2_) and KMD (O) homologous series in subnetwork 1, and primarily KMD (CH_2_) series in subnetwork 2 (Figure S12). The enrichment of KMD (CH_2_) series suggests a high presence of alkylated structures. Meanwhile, the prevalence of KMD (O) series, together with the previously observed abundance of N- containing compounds, highlight the role of heteroatom incorporation in modulating polarity.

## 4 Discussion

### 4.1 Molecular Insights into DOM Fractions from FT-ICR MS

FT-ICR MS analysis provided detailed molecular-level insights into the IHSS samples, revealing how environmental origins and extraction protocols significantly alter their molecular compositions.^68–70^ Terrestrial fractions (including both HAs and FAs) and PLFA were characterized by elevated levels of N-containing compounds, suggesting stronger microbial influence, as dissolved organic nitrogen (DON) is produced directly by microbial turnover and indirectly by extracellular enzymes released by microbes.^71,72^ The high abundance of N-containing compounds thus reflects enhanced biological transformation of these DOM fractions. Specifically, PLFA exhibited a higher abundance of aliphatic molecular compositions, highlighting its microbial-dominated origin.^73^ Due to the lack of higher plant inputs in Pony Lake, its DOM originates almost entirely from microbial sources.^74^ Accordingly, microbial exudation and cell lysis introduce membrane lipids into the DOM pool of Pony Lake, enriching it with aliphatic-rich compounds.^75,76^ In comparison, aquatic DOM was enriched in CHO compounds with higher oxygenation states, such as tannins, reflecting allochthonous inputs from terrestrial DOM, limited transformation due to short residence times, and increased photooxidation.^77,78^ Extraction methods further separated materials into different fractions based on their physiochemical properties, particularly solubility. Soil HAs, precipitated upon acidification, were characterized by aliphatic and aromatic compounds, while soil FAs, remaining in solution, contained more oxidized and polar molecules, reflecting their greater solubility. Meanwhile, aquatic HAs and FAs showed greater similarity in chemical composition, likely because IHSS uses XAD-8 resin adsorption to preferentially retain the hydrophobic fraction from aquatic materials before alkaline extraction, rather than the direct alkaline extraction employed for solid materials.^79^ It should be noted that no extraction method can fully recover the chemical nature of DOM,^80^ and our results highlight the significance of considering both environmental sources and extraction methods in the molecular characterization of DOM.

### 4.2 Morphological Profiling of DOM Fractions Using CP

Translating these molecular-level differences into functional biological outcomes remains challenging. The complexity of assessing the biological activity of DOM fractions is compounded by the limitations of conventional bioassays in resolving such mixtures and identifying bioactive components.^28^ Phenotypic screening platforms, such as the CP, provide a promising alternative by capturing subtle morphological changes that reflect cellular responses to various perturbations.^39^ While CP has proven effective in natural products research,^27,81^ its current application to DOM was hindered by issues such as strong autofluorescence and fluorescence quenching, particularly in HAs. Attempts to reduce material quantities further worsened signal loss, and current separation techniques were unable to remove the aromatic and carbohydrate components responsible for these effects.^82^ These findings underline the importance of considering intrinsic optical properties when applying fluorescence-based assays to complex mixtures like DOM.

Despite these challenges, the CP successfully captured distinct morphological profiles in response to IHSS samples. Although all samples were tested at a relatively high concentration (100 ppm), the primary objective was to induce detectable morphological changes, thereby enabling direct comparison of the bioactive potential among different samples under the same conditions and allowing integration with FT-ICR MS data to explore their potential bioactive components. The CP results revealed a clear trend in morphological activity, with increased activity observed from NOM to FAs to HAs, and from aquatic to terrestrial sources. These patterns suggest that both the extraction methods and environmental origins of DOM influence its cellular responses. The elevated activity of terrestrial HAs aligned with previous studies highlighting their antiviral properties,^61^ while challenging the assumption that a higher abundance of functional groups in FAs correlates with greater reactivity.^62^ Instead, broader molecular traits such as aromaticity and nitrogen content may play a more significant role in driving biological effects, as aromatic rings and nitrogen atoms can interact with biological targets through noncovalent interactions and hydrogen bonding, respectively.^83,84^ Exposure to terrestrial HAs led to reduced nuclear size and cell area, key features of apoptosis-like processes associated with genotoxic response.^63^ This evidence aligns with previous studies linking HAs to DNA damage and apoptotic pathways in mammalian cells,^64–66^ demonstrating the value of standardized IHSS samples in exploring the biological impacts of complex environmental mixtures. Importantly, these findings were obtained at concentrations exceeding those typically observed in natural environments, and their applicability under environmentally relevant conditions requires further validation at concentrations representative of natural systems.

### 4.3 Data Integration to Reveal Potentially Active Components in DOM Fractions

sPLS was applied to investigate the association between chemical compositions and morphological outcomes, and the first sPLS component effectively separated terrestrial HAs from other samples. Chemical features that covaried with terrestrial HAs and were associated with morphological changes clustered into two distinct regions in the van Krevelen diagram, corresponding to lipid-like compounds and BC-type condensed aromatic compounds. These classes of compounds aligned with known antiviral and bioactive fractions of humic substances, characterized by hydrophobic, aromatic-rich, and polyanionic fractions,^61,85^ emphasizing their biological significance. Mass difference and KMD analyses provided additional insight into molecular relationships and transformation pathways among these components.^67,86^ Frequent mass differences related to alkylation and redox processes, along with patterns observed in CH_2_- and O-based KMD series, highlighted the role of alkylated and oxygenated molecular compositions in influencing cellular responses. Alkylation likely increases molecular amphiphilicity and lipophilicity, thereby facilitating membrane permeability.^87^ Meanwhile, the presence of nitrogen and oxygen atoms increases the hydrogen-bonding capacity, enabling specific interactions with cellular targets including DNA or nuclear proteins.^84,88^ Consequently, the combination of high lipophilicity and strong hydrogen-bonding potential contributes to the pronounced morphological activity of terrestrial HAs, indicating that molecular characteristics rather than solely functional group counts are key in determining biological effects. Nevertheless, these results are based on sPLS correlations and high assay concentrations and should be considered as indications of potential bioactive components in IHSS samples that merit further isolation and characterization.

Nitro-polycyclic aromatic hydrocarbons (nitro-PAHs) likely occur within the BC-type condensed aromatic fraction identified by sPLS analysis. Although BC is often associated with combustion-derived sources, noncombustion pathways such as biomass oxidation can contribute substantially.^89^ Nitro-PAHs which can form via combustion, are well-known as environmental contaminants capable of inducing DNA damage,^90–92^ supporting their potential role in the observed morphological changes. These considerations make nitro-PAHs plausible contributors to the observed morphological changes. BC is an emerging pollutant of rising concern in aerosols and poses significant health risks through inhalation, linked to cardiovascular and respiratory problems, including premature death, and can exacerbate conditions like asthma.^93^ Recent studies further associate fine particles like PM2.5, which often contain BC, with cognitive decline and neurodegenerative diseases, including Alzheimer’s and Parkinson’s disease.^94–97^ Unlike physical interactions or electrostatic forces between fine particles, nitro-PAHs and derivatives may exert their effects primarily through covalent binding.^98,99^ Fine particles often carry surface charges that influence how they aggregate or interact with other components. This distinction emphasizes the importance of recognizing specific interaction mechanisms when evaluating the biological impacts of DOM. Consequently, there is a clear need for future targeted studies to identify and quantify nitro-PAHs and similar compounds within DOM fractions to better understand their roles and effects.

### 4.4 Environmental Implications

Our findings highlight specific molecular features within IHSS samples that influence cellular responses, providing insight into the potential bioactivity of DOM. While many chemical characteristics of DOM have been reported previously, the integration of high-content morphological profiling via the Cell Painting (CP) assay with statistical correlations to molecular properties offers a complementary perspective that has not been systematically explored. It is important to note that FTICR-MS is semiquantitative and biased toward strongly ionizable compounds, so the identified correlations indicate potential bioactive molecules rather than a comprehensive mapping of all bioactive species. Although FT-ICR MS intensities remain comparable across samples when preparation and analytical conditions are consistent, certain expected associations between biomolecules and morphological changes, such as those involving carbohydrate-like components, are absent in the current results. This may arise from analytical constraints (e.g., low ionization efficiency) or biological factors (e.g., limited cellular permeability). Therefore, the present work should be considered as an exploratory work, and further studies using complementary analytical techniques (e.g, Nuclear magnetic resonance) and additional multiparametric profiling assays (e.g, L1000) are encouraged to expand the application of current statistical strategy to fully explore the bioactive space of DOM.

All samples were tested at relatively high concentrations, exceeding environmentally relevant levels, to ensure measurable morphological changes. This approach was required due to the limited sensitivity of the CP assay when applied to complex environmental mixtures. Single-cell analysis is expected to improve resolution by capturing subtle and heterogeneous responses. Although the absolute magnitude of responses would likely decrease at lower concentrations, the relative bioactivity ranking remained consistent, with terrestrial humic acids exhibiting the strongest effects, particularly those associated with lipid-like compounds and nitrogen-rich black carbon–type molecules.

To prevent misinterpretation, these associations should not be taken as evidence that terrestrial humic acids in general pose health or environmental risks. Observed responses arise from specific molecular components within the IHSS samples studied. Nitro-PAH substituents are not inherent to all terrestrial humic substances, and their presence depends on environmental contamination and material processing. Established agricultural, industrial, and pharmaceutical uses of humic substances typically involve purification and quality control measures that limit such contaminants. The results therefore highlight molecular components that merit further investigation rather than indicating hazards associated with humic materials.

## Supplementary Material



## Notes

The Supporting Information provides additional methodological details and data supporting this study. A comprehensive description of the Fourier-transform ion cyclotron resonance mass spectrometry (FT-ICR MS) analysis and data processing workflow is provided in Text S1. Figure S1 illustrates the collection sites of the International Humic Substances Society (IHSS) samples. Figure S2 presents the relative abundances of heteroatom classes across IHSS samples. Figure S3 shows the results of multivariate analysis revealing compositional patterns based on FT-ICR MS data. Figure S4 shows the distributions of major compound classes in van Krevelen space. Figure S5 shows van Krevelen diagrams of representative IHSS samples. Figure S6 evaluates the autofluorescence and quenching effects of IHSS samples on Cell Painting (CP) dyes. Figure S7 outlines the workflow of the CP assay applied in this study. Figure S8 presents the ranking of morphological activities of IHSS samples. Figure S9 shows the hierarchical clustering of IHSS samples based on CP profiles. Figure S10 provides representative fluorescence images illustrating nuclear and actin/Golgi/plasma membrane compartments in control and treated cells. Figure S11 provides a network-based visualization of sparse partial least-squares (sPLS)-selected molecular features and the major mass differences observed. Figure S12 presents the Kendrick mass defect analysis of the sPLS-selected molecular features. Supplementary tables summarize the IHSS samples, fluorescent dyes and morphological features used in the CP assay, cluster assignments based on FT-ICR MS data, and mass differences applied in network analysis (Tables S1–S5) (PDF)
