# Supplementary material for: Leveraging Dissolved Organic Matter Collections as a Natural Chemical Library to Link Molecular Traits with Cellular Morphological Responses
Source: Environ Sci Technol. 2026 Mar 2;60(10):7859–71. doi: 10.1021/acs.est.5c12756 (PMC13001082; doi:10.1021/acs.est.5c12756)
Supplement: Supplementary file 1 [file es5c12756_si_001.pdf]

Supporting Information

Leveraging Dissolved Organic Matter Collections as a Natural Chemical Library to Link Molecular Traits with Cellular Morphological Responses

Xin Zhang[a], Mourad Harir[a,b,*], Joel Schick[c], Marianna Lucio[a], E. Michael Perdue[d], Philippe Schmitt-Kopplin[a,b,*]

[a]Research Unit Analytical Biogeochemistry, Helmholtz Munich, Neuherberg 85764, Germany

[b]Chair of Analytical Food Chemistry, Technical University of Munich, Freising 85354, Germany

[c]Genetics and Cellular Engineering Group, Research Unit Signaling and Translation, Helmholtz Munich, Neuherberg 85764, Germany

[d]School of Earth and Atmospheric Sciences, Georgia Institute of Technology, 311 Ferst Drive, Atlanta, Georgia, 30332-0340, United States

*Email: mourad.harir@helmholtz-munich.de.

*Email: philippe.schmittkopplin@helmholtz-munich.de.

Content:

Text S1 – Supporting Methods

Supplementary Figure S1 – Supplementary Figure S12

Supplementary Table S1 – Supplementary Table S5

Supporting information

Text S1. Fourier-transform ion cyclotron resonance mass spectrometry (FT-ICR MS) analysis and raw data processing.

Figure S1. Collecting sites for the International Humic Substances Society (IHSS) samples.

Figure S2. Relative abundances of heteroatom classes in IHSS samples.

Figure S3. Multivariate analysis of IHSS samples revealing compositional patterns based on FT-ICR MS data.

Figure S4. Van Krevelen diagram illustrates the typical regions of major compound classes.

Figure S5. Van Krevelen diagrams of representative IHSS samples selected from the four clusters identified by multivariate analysis.

Figure S6. Evaluation of autofluorescence and quenching effects of IHSS samples on CP dyes.

Figure S7. Workflow of the Cell Painting (CP) assay applied to IHSS samples.

Figure S8. Ranking of the morphological activities of IHSS samples based on their Euclidean distance to the vehicle control.

Figure S9. Hierarchical clustering of IHSS samples baes on CP profiles.

Figure S10. Representative fluorescence images illustrating nuclear and actin/Golgi/plasma membrane (AGP) compartment in control and treated cells.

Figure S11. Visualization of sPLS-selected molecular features from IHSS samples.

Figure S12. Kendrick mass defect (KMD) analysis of sPLS-selected molecular features from IHSS samples.

Table S1. IHSS samples used in this study.

Table S2. Fluorescent dyes used in the CP assay.

Table S3. List of morphological features extracted from Cell Painting images using the CellInsight™ CX7 platform.

Table S4. Cluster assignments of IHSS samples based on hierarchical clustering analysis (HCA).

Table S5. List of mass differences and corresponding elemental transformations used for network analysis. Each transformation is described by its exact mass difference and formula difference.

Supporting method

Text S1. Fourier-transform ion cyclotron resonance mass spectrometry (FT-ICR MS) analysis and raw data processing

FT-ICR MS measurements were performed using a Bruker solariX Fourier-transform ion cyclotron resonance mass spectrometer (Bremen, Germany) equipped with a 12 Tesla superconducting magnet (Magnex) and an Apollo II electrospray ionization (ESI) source operating in negative ion mode. The stock solutions prepared in DMSO were diluted with methanol to a final concentration of 70 ppm for FT-ICR MS analysis, ensuring a consistent solvent system across all samples and keeping the final DMSO content below 1%. After dilution, the samples were vortexed and centrifuged, and the resulting supernatants were introduced into the ESI source via a microliter syringe pump at a constant flow rate of 120 μL/h. The source temperature was maintained at 200 °C without nozzle-skimmer fragmentation. Ions accumulated for 300 ms prior to detection, and broadband ion excitation was applied using a frequency sweep.

Spectra were acquired under the following conditions: nebulizer gas pressure at 2.0 bar, drying gas pressure at 4.0 bar, vacuum pressure of 3×10^{-6} mbar in the quadrupole/hexapole region, and 6×10^{-10} mbar in the ICR cell. External calibration was performed using arginine cluster ions (1 mg/L in methanol). Each mass spectrum was obtained by accumulating 500 scans with a 4 megaword time domain transient. The resulting data were Fourier-transformed to the frequency domain and converted to mass spectra using Bruker's solariX Control software, covering an m/z range of 122.9 to 1000. Methanol blanks were injected between samples to minimize carryover and maintain system cleanliness.

Spectra were internally calibrated using Bruker DataAnalysis (version 5.0) and an in-house reference list of natural organic matter, achieving mass accuracy within 0.2 ppm. Calibrated spectra with a minimum signal-to-noise ratio of 4 were exported, Molecular assignment was performed using the Suwannee River Fulvic Acid (SRFA) reference list. Transformation networks were constructed from elemental mass differences derived from the assigned molecular formulas. Considered transformations included homologous series extensions ($\pm CH_2$, Δm = 14.01565 Da), hydrogenation and dehydrogenation ($\pm H_2$, Δm =

2.01565 Da), oxygen-related exchanges (±O, Δm = 15.99491 Da; ±OH, Δm = 17.00274 Da), and carbonyl-related exchanges (±CO, Δm = 27.99491 Da; $\pm CO_2$, Δm = 43.98983 Da). Nitrogen-containing transformations comprised elemental nitrogen exchange (±N, Δm = 14.00307 Da) and amination and deamination reactions (±NH, Δm = 15.01090 Da; $\pm NH_2$, Δm = 16.01872 Da), while sulfur-containing transformations included sulfur gain and loss (±S, Δm = 31.97207 Da) and sulfur oxidation ($\pm SO_3$, Δm = 79.95682 Da). Network matching was performed using a mass tolerance of 0.2 ppm, and the reference list tolerance was set to 0.2 ppm. Final elemental formulas were classified into CHO, CHNO, CHOS, and CHNOS molecular series and used to reconstruct group-selective mass spectra[1]. Shared molecular formulas were used for spectral alignment. For each sample, formula intensities were normalized to total signal intensity and used to calculate chemical indices[2]. These normalized intensities were further scaled using unit variance scaling for multivariate analysis.

Chemical indices including the double bond equivalent (DBE), carbon-normalized DBE (DBE/C), and the modified aromaticity index (AImod) were calculated using Eqs.1 and 2 to assess the degree of unsaturation and aromaticity of the assigned molecular formulas[3].

$$DBE = 1 + \frac{1}{2}(2C - H + N) \qquad \text{Eq.1}$$

$$AI_{mod} = \frac{1 + C - \frac{1}{2}O - S - \frac{1}{2}(H + N)}{C - \frac{1}{2}O - N - S} \qquad \text{Eq.2}$$

Where C, H, O, N and S represent the number of carbon, hydrogen, oxygen, nitrogen, and sulfur atoms in the assigned formulas.

Intensity-weighted averages of chemical indices (Xw) were calculated for each sample using Eq.3[4]:

$$X_{wa} = \frac{\sum_{i=1}^{n} X_i \times Int_i}{\sum_{i=1}^{n} Int_i} \qquad \text{Eq.3}$$

Where Xi represents the value of the chemical index for formula i, Inti represents the corresponding intensity, and n is the total number of assigned formulas in the sample.

FT-ICR MS data were primarily visualized using van Krevelen and mass-edited H/C ratio diagrams. Van Krevelen diagrams were constructed by plotting H/C ratio against O/C ratio for each formula, facilitating the identification of compound classes based on their positions within the plot[5]. Mass-edited H/C ratio diagrams were generated by plotting H/C ratio against the molecular weight of corresponding neutral mass, providing insights into the distribution of molecular saturation and molecular weight. In addition, the mass difference network was constructed by matching the exact mass differences among all selected molecules with a predefined list of 40 mass differences corresponding to common functional groups and molecular transformations (Table S5). Observed formula differences were then assigned to specific chemical transformations. For example, addition of CO corresponded to carbonylation, while +C indicated carbon addition or condensation. Dicarbonylation was represented by $+C_2O_2$ and $+C_3H_2O_2$, whereas oxygenation, hydroxylation, or carbonylation corresponded to +O. Alkylation was indicated by +CH2 and $+C_2H_4$, and acylation by $+C_2H_2O$, $+C_3H_4O$, and $+C_4H_4O_2$. Unsaturation was represented by $+C_2H_2$, hydroxymethylation or formylation by $+CH_2O$, and hydrogenation or dehydrogenation by $+H_2$. Oxidation was assigned to $+O_2$, O-alkylation to $+C_2H_4O$ and $+C_4H_8O$, unsaturated acylation to $+C_4H_6O$, deoxygenative alkylation to $–O+C_3H_6$, carboxylation to $+CO_2$, and oxidative cleavage to $–CH_2+O$, following the approach in our previous study[1], and KMD values for different chemical groups were calculated using E.4 and Eq.5[6]:

$$Kendrick\ mass\ (X) = calculated\ mass\ \times \frac{nominal\ mass\ of\ X}{exact\ mass\ of\ X} \qquad \text{Eq.4}$$

$$KMD(X) = nominal\ Kenrick\ mass\ (X) - Kendrick\ mass\ (X) \qquad \text{Eq.5}$$

Where X represents the selected group.

Supplementary Figures

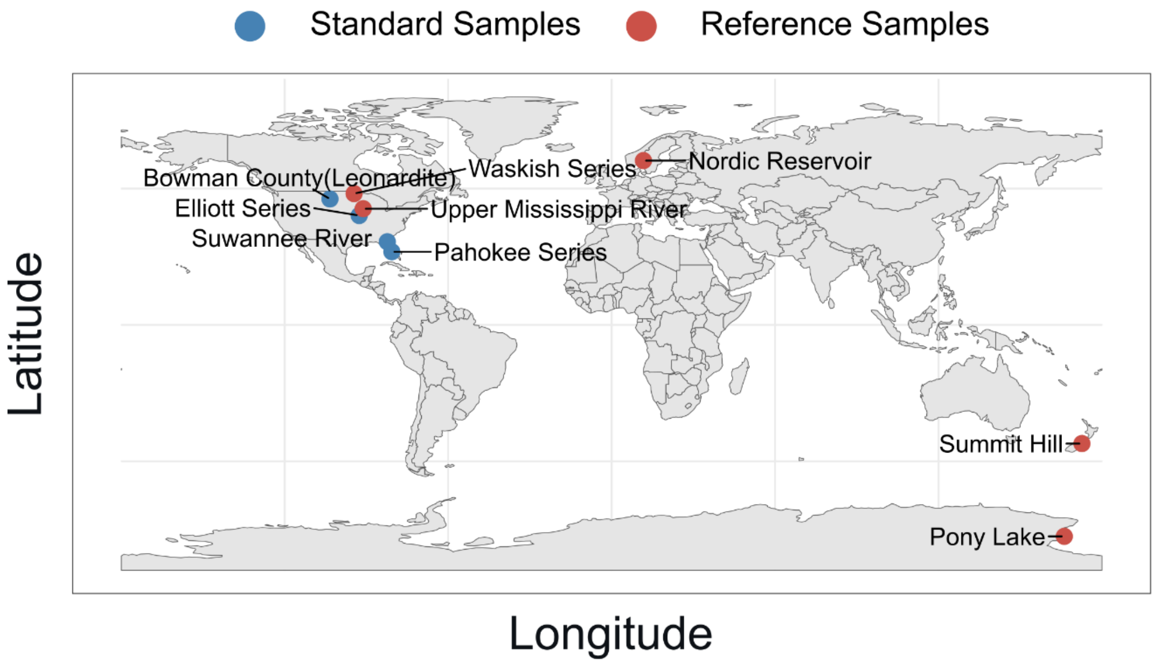

Figure S1. Global distribution of collection sites for International Humic Substances Society (IHSS) source materials. Standard samples, which were collected and prepared following all IHSS protocols, are shown in blue; Reference samples, which did not meet all IHSS criteria during collection or preparation, are shown in red.

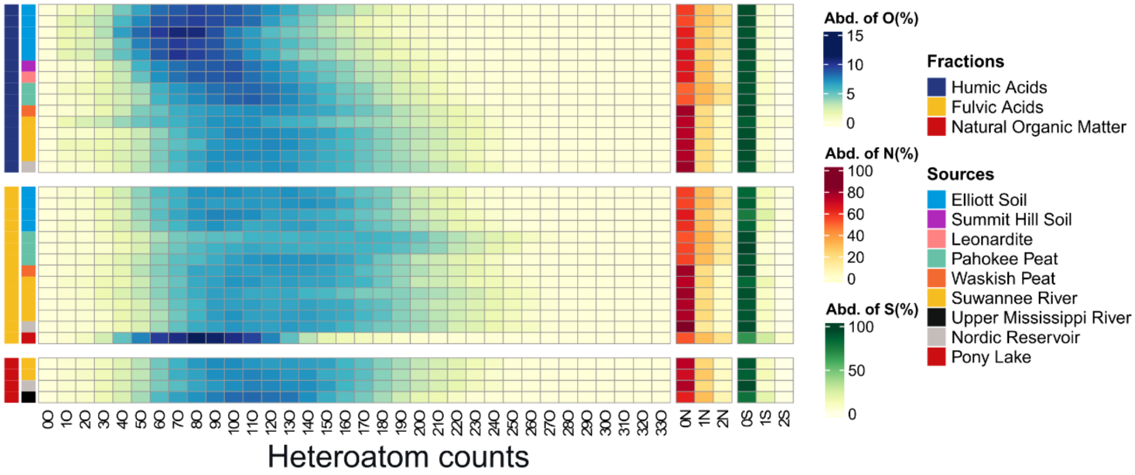

Figure S2. Relative abundances of heteroatom classes in IHSS samples, based on the number of oxygen (O), nitrogen (N), and sulfur (S) atoms in formulas assigned by Fourier-transform ion cyclotron resonance mass spectrometry (FT-ICR MS) analysis. Each heatmap corresponds to one heteroatom type (O, N, or S), with the horizontal dimension showing the heteroatom counts per molecular formula. Samples are grouped by fractions and sources, as indicated by the color bars on the left. The color scale represents the relative abundance (%) of formulas containing the corresponding heteroatom count.

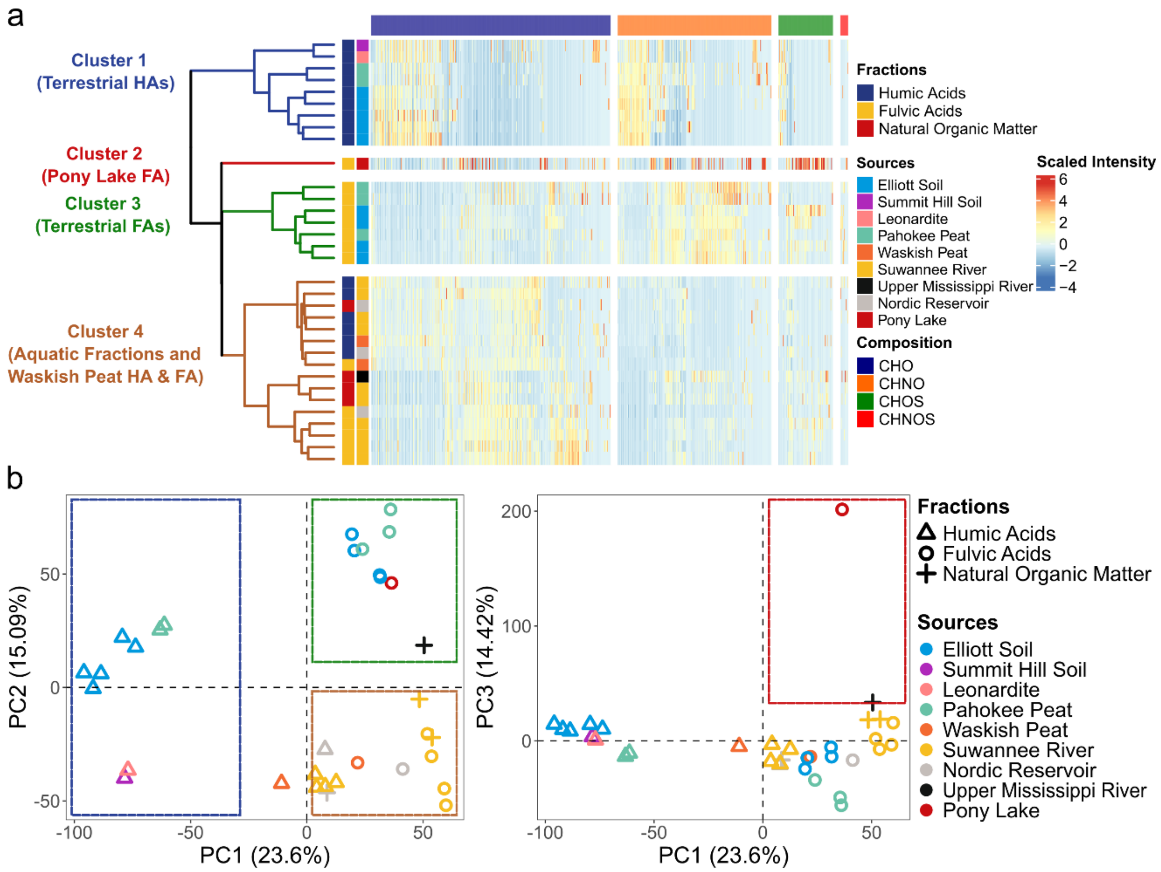

Figure S3. Multivariate analysis of IHSS samples revealing compositional patterns based on FT-ICR MS data. (a) Hierarchical clustering analysis (HCA) of IHSS samples based on assigned molecular formulas, with formulas grouped by elemental composition. Four clusters are indicated in the dendrogram: Cluster 1 (Terrestrial HAs), Cluster 2 (Pony Lake FA), Cluster 3 (Terrestrial FAs), and Cluster 4 (Aquatic fractions and Waskish Peat HA & FA). (b) Principal component analysis (PCA) showing sample distributions along principal component one and two (left panel) and principal component one and three (right panel). Dashed boxes highlight the PCA groupings corresponding to the HCA clusters: the blue box corresponds to Cluster 1, the red box to Cluster 2, the green box to Cluster 3, and the yellow box to Cluster 4. Samples are labeled by both color and shape, with color indicating the source materials and shape indicating the IHSS fractions.

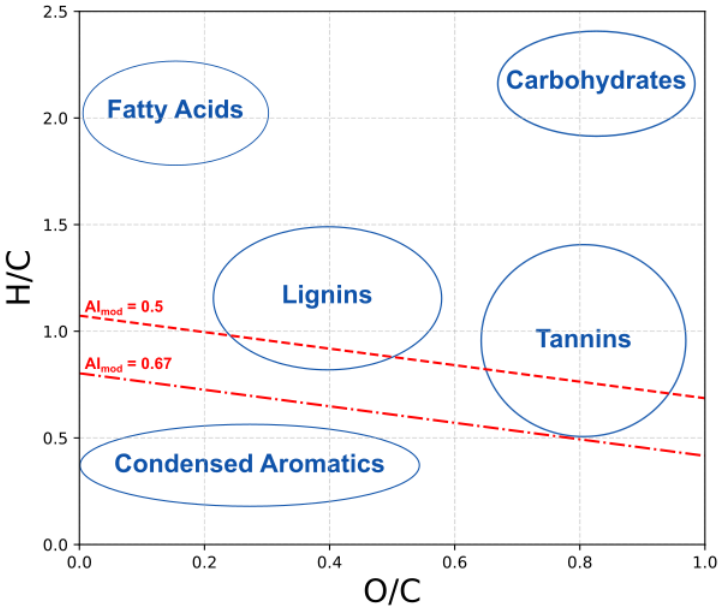

Figure S4. Van Krevelen diagram illustrating the typical regions of major compound classes. Blue circles indicate the characteristic locations of different compound classes. Red dashed and dash-dotted lines represent modified aromaticity index (AI_{mod}) at 0.5 and 0.67, which distinguish aromatic and condensed compounds, respectively.

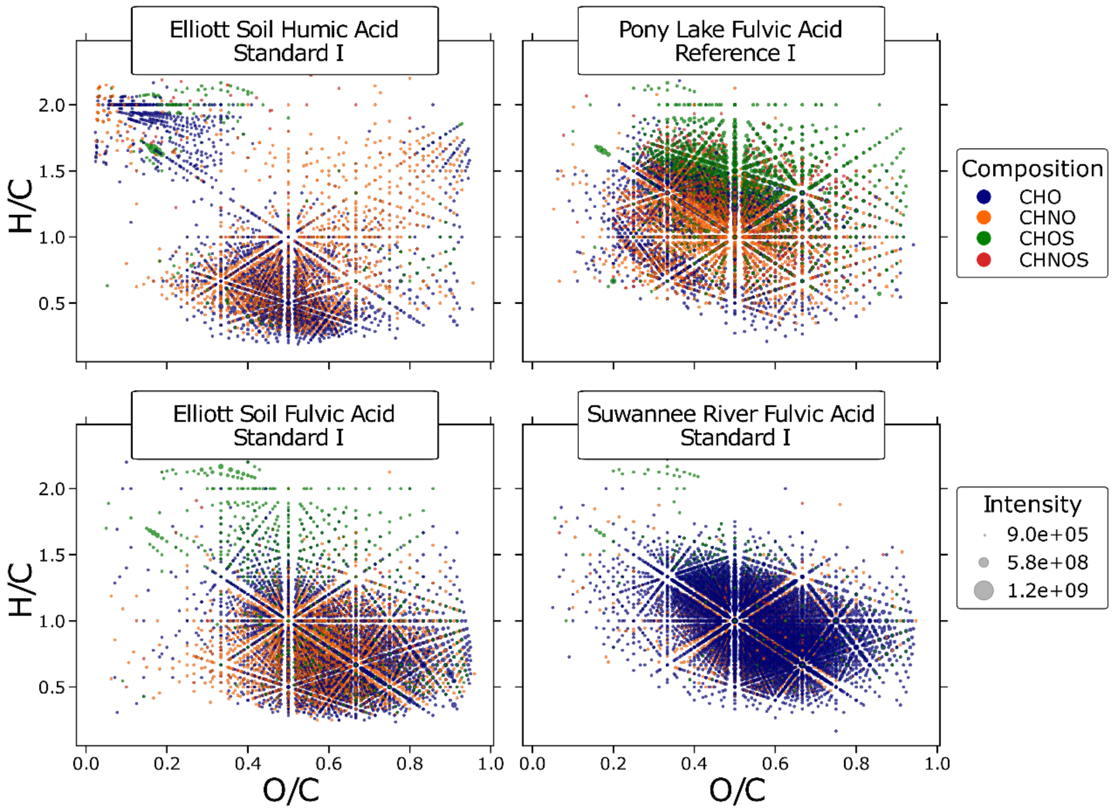

Figure S5. Van Krevelen diagrams of representative IHSS samples selected from the four clusters identified by multivariate analysis. Elliot Soil Humic Acid Standard I was selected from the first cluster (Terrestrial Humic Acids); Pony Lake Fulvic Acid Reference I constitutes the second cluster; Elliot Soil Fulvic Acid Standard I was selected from the third cluster (Terrestrial Fulvic Acids); and Suwannee River Fulvic Acid Standard I was selected from the fourth cluster (Aquatic fractions, Waskish Peat Humic Acid and Fulvic Acid). Bubbles are color-coded according to CHO (blue), CHNO (orange), CHOS (green), and CHNOS (red). Bubble sizes represent the relative signal intensities of mass peaks.

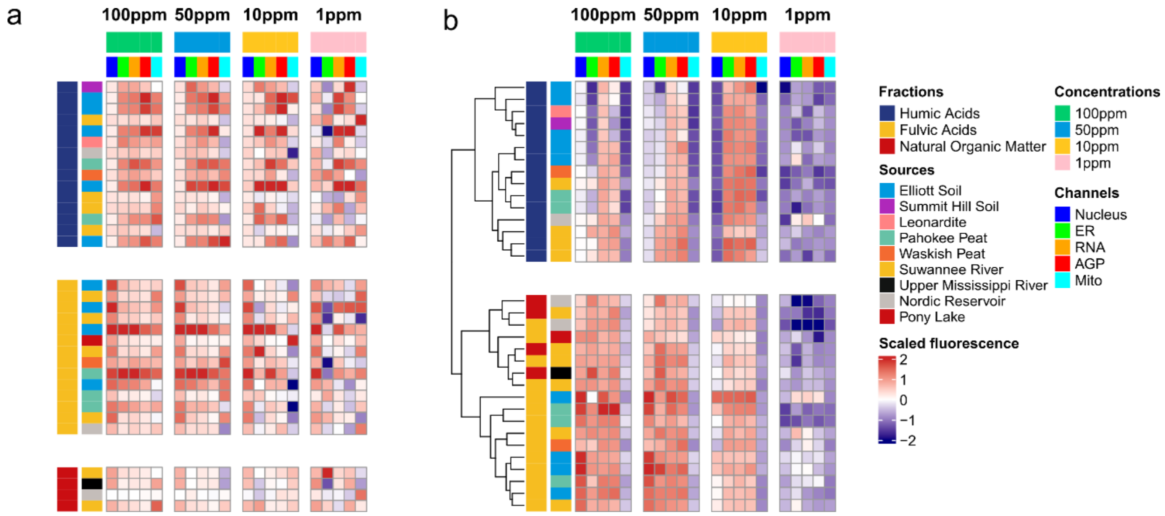

Figure S6. Evaluation of autofluorescence and quenching effects of IHSS samples on CP dyes. (a) Heatmap showing the fluorescence of IHSS samples across five imaging channels corresponding to the CP at four concentrations (100, 50, 10, and 1 ppm).Fluorescence intensities were subtracted by the signal from phosphate-buffered saline (PBS) to quantify sample-derived autofluoresence. Positive values (red) indicate higher autofluorescence, while near-zero (white) or negative values (blue) indicate negligible or below-background fluorescence. (b) Heatmap showing hierarchical clustering of fluorescence differences between dye-sample mixtures and dyes alone under corresponding conditions, indicating the extent of fluorescence quenching induced by the samples. Negative values (blue) indicate sample-induced quenching of dye fluoresence, whereas positive values (red) indicate signal enhancement, likely due to autofluorescence of samples.

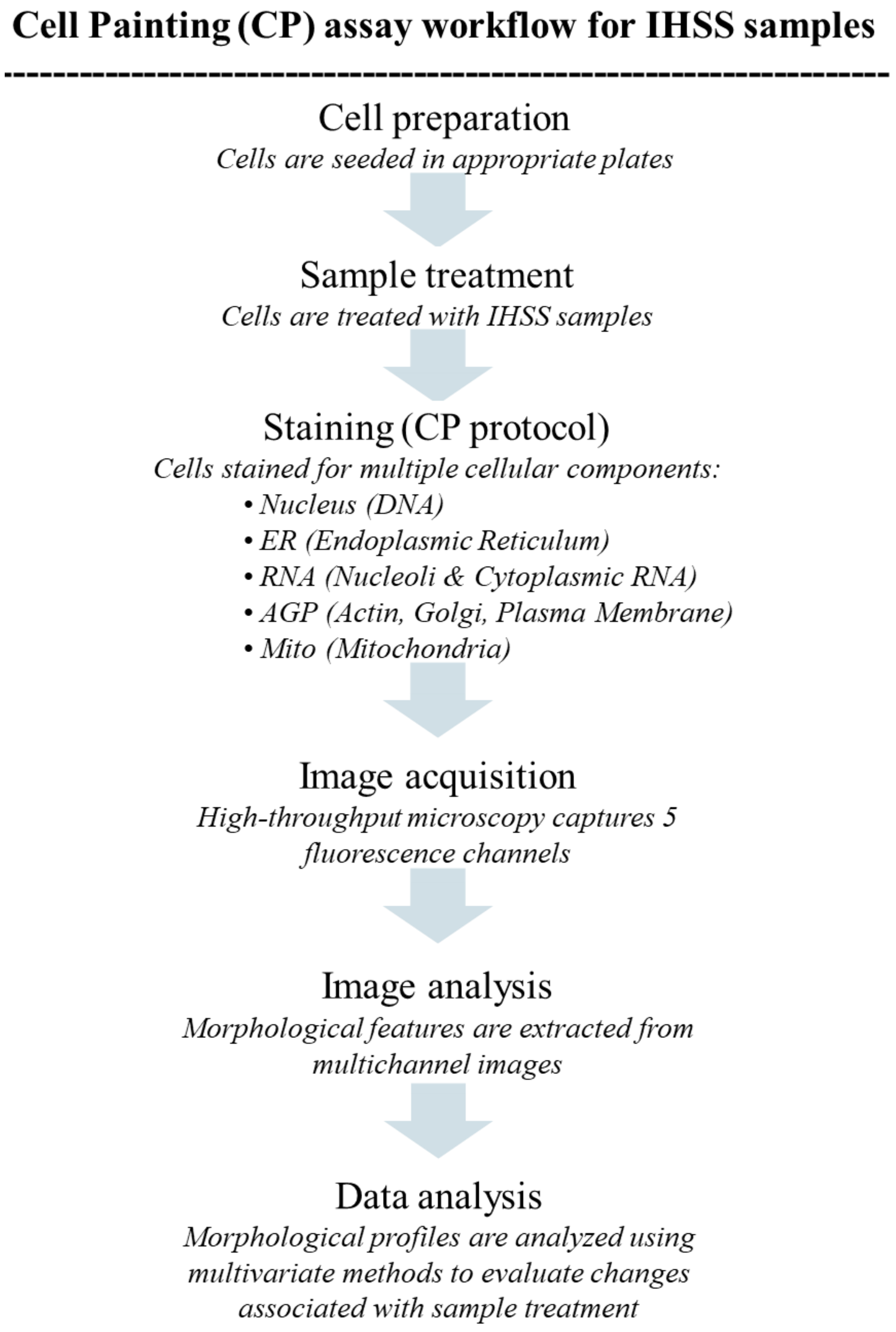

Figure S7. Workflow of the Cell Painting (CP) Assay for IHSS Samples.

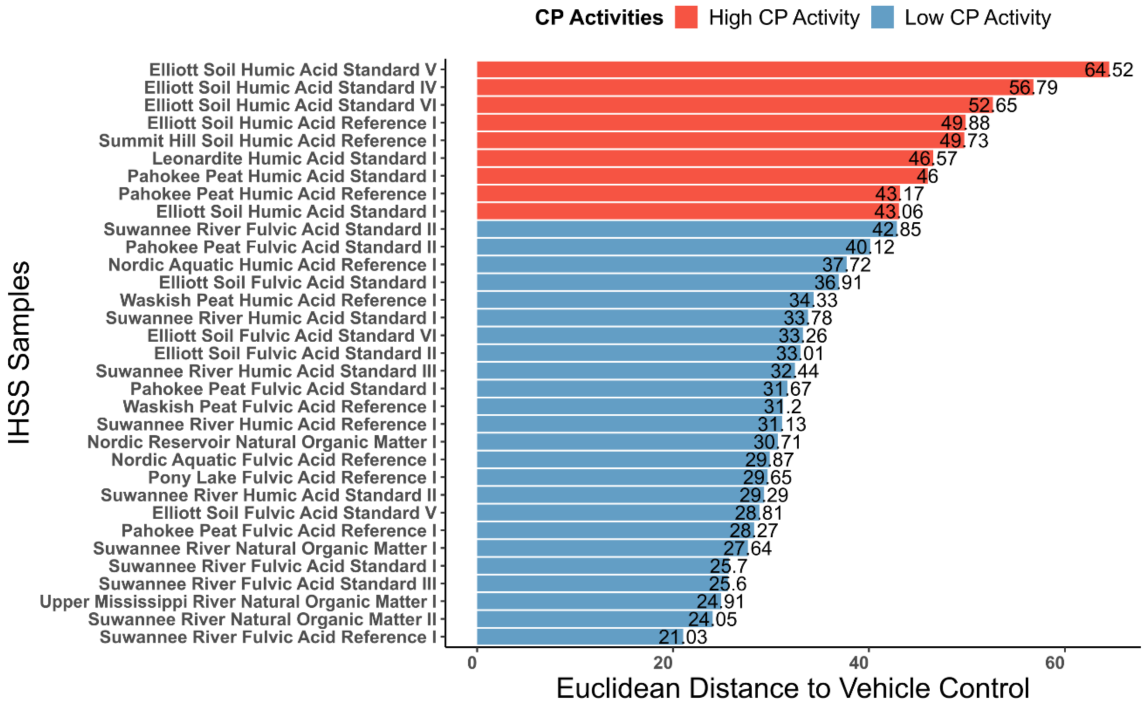

Figure S8. Ranking of the morphological activities of IHSS samples based on their Euclidean distance to the vehicle control. The x-axis represents the Euclidean distance derived from CP morphological profiles, indicating the extent of phenotypic changes from untreated cells. Sample with higher CP activity (labeled in red) induce stronger morphological changes, while those with lower CP activity (labeled in blue) exhibit weaker morphological activities.

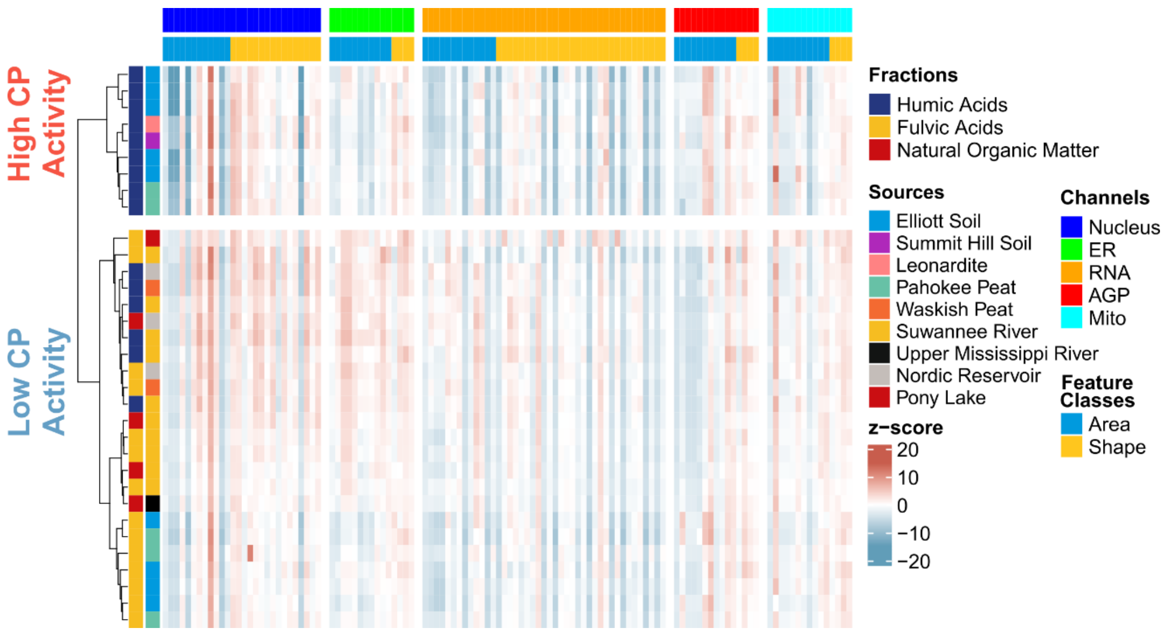

Figure S9. Hierarchical clustering of IHSS samples baes on CP profiles. Samples were clustered using median morphological profiles derived from six replicates per treatment. Clusters are annotated by CP activity, with high-activity samples (red) exhibiting stronger morphological changes and low-activity samples (blue) showing milder morphological effects. Annotation bars indicate the fraction, sample source, imaging channels, and feature classes. The heatmap shows normalized morphological features and expressed as z-score.

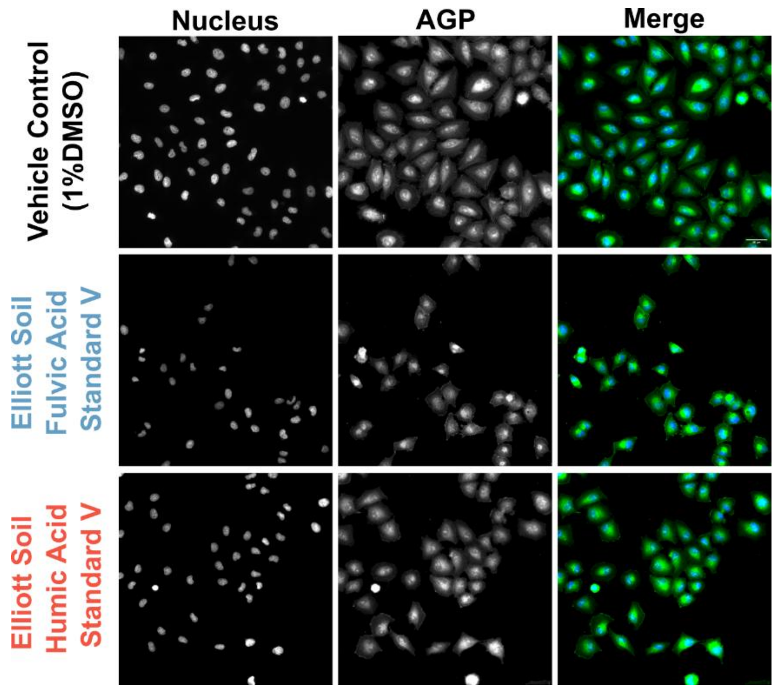

Figure S10. Representative fluorescence images illustrating nuclear and actin/Golgi/plasma membrane (AGP) compartment in control and treated cells. The blue-labeled sample represents a low-CP-activity treatment that induces relatively mild morphological changes, whereas the red-labeled sample represents a high-CP-activity treatment that elicits stronger morphological changes. All images were acquired at 20× magnification. Scale bar: 20 micrometers.

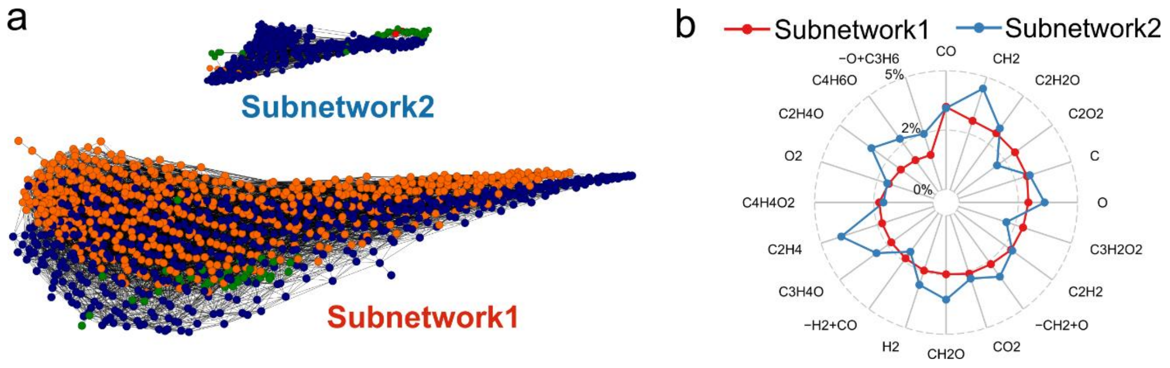

Figure S11. Visualization of sPLS-selected molecular features from IHSS samples. (a) Mass difference network constructed from the sPLS-selected molecular formulas using a predefined list of mass differences corresponding to common functional groups and molecular transformations. Nodes are colored according to molecular class: CHO (blue), CHNO (orange), and CHOS (green). Two distinct clusters are observed and labeled as Subnetwork 1 and Subnetwork 2, representing distinct molecular families. (b) Radar plot summarizing the most frequent mass differences observed within each subnetwork, with Subnetwork 1 shown in red and Subnetwork 2 shown in blue, illustrating the characteristic mass differences that dominate each cluster. The dominant mass-difference units correspond to CH_2-derived alkyl units (e.g., CH_2, C_2H_4) and redox-related changes involving oxygen (e.g., CO, O), suggesting that alkylation and redox processes are the key transformations within both subnetworks.

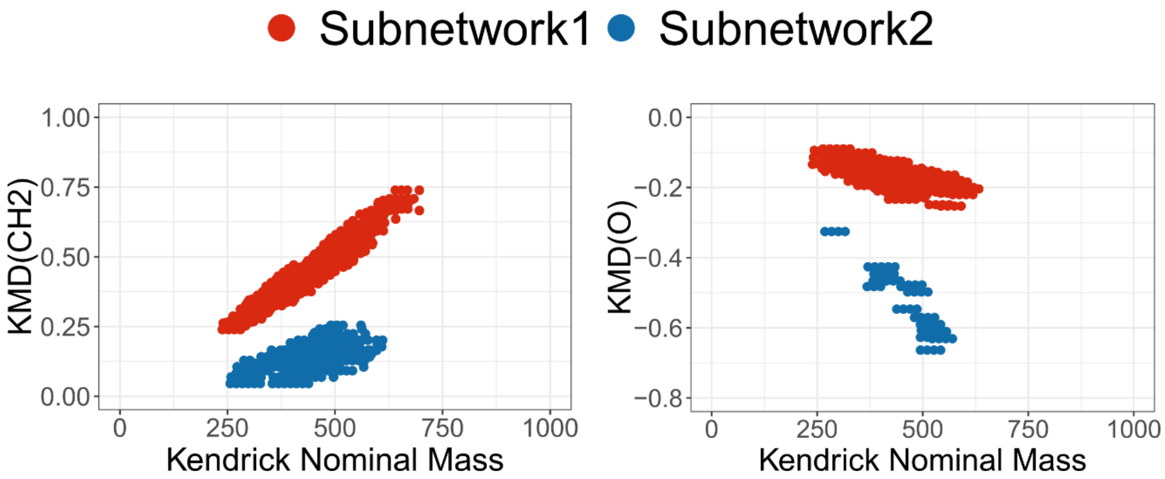

Figure S12. Kendrick mass defect (KMD) analysis of sPLS-selected molecular features from IHSS samples. KMD plots showing CH2 and O homologous series identified within subnetworks derived from sPLS-selected features. Only homologous containing at least three members are shown.

Supplementary Tables

Table S1. International Humic Substances Society (IHSS) samples used in this study.

Sample	IHSS Cat.No.	Sources	Fractions
Leonardite Humic Acid Standard I	1S104H	Leonardite	Humic Acid
Elliott Soil Humic Acid Standard I	1S102H	Elliott Soil	Humic Acid
Elliott Soil Humic Acid Standard IV	4S102H	Elliott Soil	Humic Acid
Elliott Soil Humic Acid Standard V	5S102H	Elliott Soil	Humic Acid
Elliott Soil Humic Acid Standard VI	6S102H	Elliott Soil	Humic Acid
Pahokee Peat Humic Acid Standard I	1S103H	Pahokee Peat	Humic Acid
Suwannee River Humic Acid Standard I	1S101H	Suwannee River	Humic Acid
Suwannee River Humic Acid Standard II	2S101H	Suwannee River	Humic Acid
Suwannee River Humic Acid Standard III	3S101H	Suwannee River	Humic Acid
Elliott Soil Humic Acid Reference I	1R102H	Elliott Soil	Humic Acid
Summit Hill Soil Humic Acid Reference I	1R106H	Summit Hill Soil	Humic Acid
Pahokee Peat Humic Acid Reference I	1R103H	Pahokee Peat	Humic Acid
Waskish Peat Humic Acid Reference I	1R107H	Waskish Peat	Humic Acid
Suwannee River Humic Acid Reference I	1R101H	Suwannee River	Humic Acid
Nordic Aquatic Humic Acid Reference I	1R105H	Nordic Reservoir	Humic Acid
Elliott Soil Fulvic Acid Standard I	1S102F	Elliott Soil	Fulvic Acid
Elliott Soil Fulvic Acid Standard II	2S102F	Elliott Soil	Fulvic Acid
Elliott Soil Fulvic Acid Standard V	5S102F	Elliott Soil	Fulvic Acid
Elliott Soil Fulvic Acid Standard VI	6S102F	Elliott Soil	Fulvic Acid
Pahokee Peat Fulvic Acid Standard I	1S103F	Pahokee Peat	Fulvic Acid

Pahokee Peat Fulvic Acid Standard II	2S103F	Pahokee Peat	Fulvic Acid
Suwannee River Fulvic Acid Standard I	1S101F	Suwannee River	Fulvic Acid
Suwannee River Fulvic Acid Standard II	2S101F	Suwannee River	Fulvic Acid
Suwannee River Fulvic Acid Standard III	3S101F	Suwannee River	Fulvic Acid
Pahokee Peat Fulvic Acid Reference I	1R103F	Pahokee Peat	Fulvic Acid
Waskish Peat Fulvic Acid Reference I	1R107F	Waskish Peat	Fulvic Acid
Suwannee River Fulvic Acid Reference I	1R101F	Suwannee River	Fulvic Acid
Nordic Aquatic Fulvic Acid Reference I	1R105F	Nordic Reservoir	Fulvic Acid
Pony Lake Fulvic Acid Reference I	1R109F	Pony Lake	Fulvic Acid
Suwannee River Natural Organic Matter I	1R101N	Suwannee River	Natural Organic Matter
Suwannee River Natural Organic Matter II	2R101N	Suwannee River	Natural Organic Matter
Nordic Reservoir Natural Organic Matter I	1R108N	Nordic Reservoir	Natural Organic Matter
Upper Mississipi River Natural Organic Matter I	1R110N	Mississippi River	Natural Organic Matter

Table S2. Fluorescent dyes used in the Cell Painting assay.

Cell painting Dyes	Catalogue Number	Opera phenix		Organelle or cellular components
		Ex. (nm)	*Em. (nm)*	
Hoechst 33342	H3570	350	461	Nucleus
Concanavalin A	C11252	495	519	Endoplasmic reticulum
SYTO 14 green fluorescent nucleic acid stain	S7576	517(DNA), 521(RNA)	549(DNA), 547(RNA)	Nucleoli, cytoplasmic RNA
Wheat Germ Agglutinin/Alexa Fluor 555 conjugate	W32464	555	565	Golgi, plasma membrane
Phalloidin/Alexa Fluor 568 conjugate	A12380	578	600	F-actin, cytoskeleton
Mito Tracker Deep Red	M22426	644	665	Mitochondria

Table S3. List of morphological features extracted from Cell Painting images using the CellInsight™ CX7 platform.

Morphological features	Analysis module (BioApplication)	Imaging channel	Feature category
spot_ValidObjectCount	*Compartmental Analysis*	*Nucleus*	*Count*
spot_ObjectTotalArea_Nucleus	*Compartmental Analysis*	Nucleus	Area
spot_ObjectAvgArea_Nucleus	*Compartmental Analysis*	Nucleus	Area
spot_ObjectTotalInten_Nucleus	*Compartmental Analysis*	*Nucleus*	*Intensity*
spot_ObjectAvgInten_Nucleus	*Compartmental Analysis*	*Nucleus*	*Intensity*
spot_ObjectTotalIntenPerObject_Nucleus	*Compartmental Analysis*	*Nucleus*	*Intensity*
spot_MEAN_ObjectArea_Nucleus	*Compartmental Analysis*	Nucleus	Area
spot_SD_ObjectArea_Nucleus	*Compartmental Analysis*	Nucleus	Area
spot_MEAN_ObjectShapeP2A_Nucleus	*Compartmental Analysis*	Nucleus	Shape
spot_SD_ObjectShapeP2A_Nucleus	*Compartmental Analysis*	Nucleus	Shape
spot_MEAN_ObjectShapeLWR_Nucleus	*Compartmental Analysis*	Nucleus	Shape
spot_SD_ObjectShapeLWR_Nucleus	*Compartmental Analysis*	Nucleus	Shape
spot_MEAN_ObjectTotalInten_Nucleus	*Compartmental Analysis*	*Nucleus*	*Intensity*

spot_SD_ObjectTotalInten_Nucleus	*Compartmental Analysis*	*Nucleus*	*Intensity*
spot_MEAN_ObjectAvgInten_Nucleus	*Compartmental Analysis*	*Nucleus*	*Intensity*
spot_SD_ObjectAvgInten_Nucleus	*Compartmental Analysis*	*Nucleus*	*Intensity*
spot_MEAN_ObjectVarInten_Nucleus	*Compartmental Analysis*	*Nucleus*	*Intensity*
spot_SD_ObjectVarInten_Nucleus	*Compartmental Analysis*	*Nucleus*	*Intensity*
spot_SpotCount_ER	*Compartmental Analysis*	*Endoplasmic reticulum*	*Count*
spot_SpotTotalArea_ER	*Compartmental Analysis*	Endoplasmic reticulum	Area
spot_SpotAvgArea_ER	*Compartmental Analysis*	Endoplasmic reticulum	Area
spot_SpotTotalInten_ER	*Compartmental Analysis*	*Endoplasmic reticulum*	*Intensity*
spot_SpotAvgInten_ER	*Compartmental Analysis*	*Endoplasmic reticulum*	*Intensity*
spot_SpotTotalIntenPerSpot_ER	*Compartmental Analysis*	*Endoplasmic reticulum*	*Intensity*
spot_SpotCountPerObject_ER	*Compartmental Analysis*	*Endoplasmic reticulum*	*Count*
spot_SpotTotalAreaPerObject_ER	*Compartmental Analysis*	Endoplasmic reticulum	Area
spot_SpotTotalIntenPerObject_ER	*Compartmental Analysis*	*Endoplasmic reticulum*	*Intensity*
spot_MEAN_ObjectSpotTotalCount_ER	*Compartmental Analysis*	*Endoplasmic reticulum*	*Count*

spot_SD_ObjectSpotTotalCount_ER	*Compartmental Analysis*	*Endoplasmic reticulum*	*Count*
spot_MEAN_ObjectSpotTotalArea_ER	*Compartmental Analysis*	Endoplasmic reticulum	Area
spot_SD_ObjectSpotTotalArea_ER	*Compartmental Analysis*	Endoplasmic reticulum	Area
spot_MEAN_ObjectSpotAvgArea_ER	*Compartmental Analysis*	Endoplasmic reticulum	Area
spot_SD_ObjectSpotAvgArea_ER	*Compartmental Analysis*	Endoplasmic reticulum	Area
spot_MEAN_ObjectSpotTotalInten_ER	*Compartmental Analysis*	*Endoplasmic reticulum*	*Intensity*
spot_SD_ObjectSpotTotalInten_ER	*Compartmental Analysis*	*Endoplasmic reticulum*	*Intensity*
spot_MEAN_ObjectSpotAvgInten_ER	*Compartmental Analysis*	*Endoplasmic reticulum*	*Intensity*
spot_SD_ObjectSpotAvgInten_ER	*Compartmental Analysis*	*Endoplasmic reticulum*	*Intensity*
spot_SpotCount_Nucleoli_RNA	*Compartmental Analysis*	*Nucleoli and cytoplasmic RNA*	*Count*
spot_SpotTotalArea_Nucleoli_RNA	*Compartmental Analysis*	Nucleoli and cytoplasmic RNA	Area
spot_SpotAvgArea_Nucleoli_RNA	*Compartmental Analysis*	Nucleoli and cytoplasmic RNA	Area
spot_SpotTotalInten_Nucleoli_RNA	*Compartmental Analysis*	*Nucleoli and cytoplasmic RNA*	*Intensity*
spot_SpotAvgInten_Nucleoli_RNA	*Compartmental Analysis*	*Nucleoli and cytoplasmic RNA*	*Intensity*
spot_SpotTotalIntenPerSpot_Nucleoli_RNA	*Compartmental Analysis*	*Nucleoli and cytoplasmic RNA*	*Intensity*

spot_SpotCountPerObject_Nucleoli_RNA	*Compartmental Analysis*	*Nucleoli and cytoplasmic RNA*	*Count*
spot_SpotTotalAreaPerObject_Nucleoli_RNA	*Compartmental Analysis*	Nucleoli and cytoplasmic RNA	Area
spot_SpotTotalIntenPerObject_Nucleoli_RNA	*Compartmental Analysis*	*Nucleoli and cytoplasmic RNA*	*Intensity*
spot_MEAN_ObjectSpotTotalCount_Nucleoli_RNA	*Compartmental Analysis*	*Nucleoli and cytoplasmic RNA*	*Count*
spot_SD_ObjectSpotTotalCount_Nucleoli_RNA	*Compartmental Analysis*	*Nucleoli and cytoplasmic RNA*	*Count*
spot_MEAN_ObjectSpotTotalArea_Nucleoli_RNA	*Compartmental Analysis*	Nucleoli and cytoplasmic RNA	Area
spot_SD_ObjectSpotTotalArea_Nucleoli_RNA	*Compartmental Analysis*	Nucleoli and cytoplasmic RNA	Area
spot_MEAN_ObjectSpotAvgArea_Nucleoli_RNA	*Compartmental Analysis*	Nucleoli and cytoplasmic RNA	Area
spot_SD_ObjectSpotAvgArea_Nucleoli_RNA	*Compartmental Analysis*	Nucleoli and cytoplasmic RNA	Area
spot_MEAN_ObjectSpotTotalInten_Nucleoli_RNA	*Compartmental Analysis*	*Nucleoli and cytoplasmic RNA*	*Intensity*
spot_SD_ObjectSpotTotalInten_Nucleoli_RNA	*Compartmental Analysis*	*Nucleoli and cytoplasmic RNA*	*Intensity*
spot_MEAN_ObjectSpotAvgInten_Nucleoli_RNA	*Compartmental Analysis*	*Nucleoli and cytoplasmic RNA*	*Intensity*
spot_SD_ObjectSpotAvgInten_Nucleoli_RNA	*Compartmental Analysis*	*Nucleoli and cytoplasmic RNA*	*Intensity*
spot_SpotCount_AGP	*Compartmental Analysis*	*Actin, Golgi, and plasma membrane*	*Count*
spot_SpotTotalArea_AGP	*Compartmental Analysis*	Actin, Golgi, and plasma membrane	Area

spot_SpotAvgArea_AGP	*Compartmental Analysis*	Actin, Golgi, and plasma membrane	Area
spot_SpotTotalInten_AGP	*Compartmental Analysis*	*Actin, Golgi, and plasma membrane*	*Intensity*
spot_SpotAvgInten_AGP	*Compartmental Analysis*	*Actin, Golgi, and plasma membrane*	*Intensity*
spot_SpotTotalIntenPerSpot_AGP	*Compartmental Analysis*	*Actin, Golgi, and plasma membrane*	*Intensity*
spot_SpotCountPerObject_AGP	*Compartmental Analysis*	*Actin, Golgi, and plasma membrane*	*Count*
spot_SpotTotalAreaPerObject_AGP	*Compartmental Analysis*	Actin, Golgi, and plasma membrane	Area
spot_SpotTotalIntenPerObject_AGP	*Compartmental Analysis*	*Actin, Golgi, and plasma membrane*	*Intensity*
spot_MEAN_ObjectSpotTotalCount_AGP	*Compartmental Analysis*	*Actin, Golgi, and plasma membrane*	*Count*
spot_SD_ObjectSpotTotalCount_AGP	*Compartmental Analysis*	*Actin, Golgi, and plasma membrane*	*Count*
spot_MEAN_ObjectSpotTotalArea_AGP	*Compartmental Analysis*	Actin, Golgi, and plasma membrane	Area
spot_SD_ObjectSpotTotalArea_AGP	*Compartmental Analysis*	Actin, Golgi, and plasma membrane	Area
spot_MEAN_ObjectSpotAvgArea_AGP	*Compartmental Analysis*	Actin, Golgi, and plasma membrane	Area
spot_SD_ObjectSpotAvgArea_AGP	*Compartmental Analysis*	Actin, Golgi, and plasma membrane	Area
spot_MEAN_ObjectSpotTotalInten_AGP	*Compartmental Analysis*	*Actin, Golgi, and plasma membrane*	*Intensity*
spot_SD_ObjectSpotTotalInten_AGP	*Compartmental Analysis*	*Actin, Golgi, and plasma membrane*	*Intensity*

spot_MEAN_ObjectSpotAvgInten_AGP	*Compartmental Analysis*	*Actin, Golgi, and plasma membrane*	*Intensity*
spot_SD_ObjectSpotAvgInten_AGP	*Compartmental Analysis*	*Actin, Golgi, and plasma membrane*	*Intensity*
spot_SpotCount_Mito	*Compartmental Analysis*	*Mitochondria*	*Count*
spot_SpotTotalArea_Mito	*Compartmental Analysis*	Mitochondria	Area
spot_SpotAvgArea_Mito	*Compartmental Analysis*	Mitochondria	Area
spot_SpotTotalInten_Mito	*Compartmental Analysis*	*Mitochondria*	*Intensity*
spot_SpotAvgInten_Mito	*Compartmental Analysis*	*Mitochondria*	*Intensity*
spot_SpotTotalIntenPerSpot_Mito	*Compartmental Analysis*	*Mitochondria*	*Intensity*
spot_SpotCountPerObject_Mito	*Compartmental Analysis*	*Mitochondria*	*Count*
spot_SpotTotalAreaPerObject_Mito	*Compartmental Analysis*	Mitochondria	Area
spot_SpotTotalIntenPerObject_Mito	*Compartmental Analysis*	*Mitochondria*	*Intensity*
spot_MEAN_ObjectSpotTotalCount_Mito	*Compartmental Analysis*	*Mitochondria*	*Count*
spot_SD_ObjectSpotTotalCount_Mito	*Compartmental Analysis*	*Mitochondria*	*Count*
spot_MEAN_ObjectSpotTotalArea_Mito	*Compartmental Analysis*	Mitochondria	Area
spot_SD_ObjectSpotTotalArea_Mito	*Compartmental Analysis*	Mitochondria	Area

spot_MEAN_ObjectSpotAvgArea_Mito	*Compartmental Analysis*	Mitochondria	Area
spot_SD_ObjectSpotAvgArea_Mito	*Compartmental Analysis*	Mitochondria	Area
spot_MEAN_ObjectSpotTotalInten_Mito	*Compartmental Analysis*	*Mitochondria*	*Intensity*
spot_SD_ObjectSpotTotalInten_Mito	*Compartmental Analysis*	*Mitochondria*	*Intensity*
spot_MEAN_ObjectSpotAvgInten_Mito	*Compartmental Analysis*	*Mitochondria*	*Intensity*
spot_SD_ObjectSpotAvgInten_Mito	*Compartmental Analysis*	*Mitochondria*	*Intensity*
mor_MEAN_ObjectArea_Nucleoli_RNA	*Morphology Explorer*	Nucleoli and cytoplasmic RNA	Area
mor_SD_ObjectArea_Nucleoli_RNA	*Morphology Explorer*	Nucleoli and cytoplasmic RNA	Area
mor_MEAN_ObjectPerim_Nucleoli_RNA	*Morphology Explorer*	Nucleoli and cytoplasmic RNA	Shape
mor_SD_ObjectPerim_Nucleoli_RNA	*Morphology Explorer*	Nucleoli and cytoplasmic RNA	Shape
mor_MEAN_ObjectShapeP2A_Nucleoli_RNA	*Morphology Explorer*	Nucleoli and cytoplasmic RNA	Shape
mor_SD_ObjectShapeP2A_Nucleoli_RNA	*Morphology Explorer*	Nucleoli and cytoplasmic RNA	Shape
mor_MEAN_ObjectShapeLWR_Nucleoli_RNA	*Morphology Explorer*	Nucleoli and cytoplasmic RNA	Shape
mor_SD_ObjectShapeLWR_Nucleoli_RNA	*Morphology Explorer*	Nucleoli and cytoplasmic RNA	Shape
mor_MEAN_ObjectShapeBFR_Nucleoli_RNA	*Morphology Explorer*	Nucleoli and cytoplasmic RNA	Shape

mor_SD_ObjectShapeBFR_Nucleoli_RNA	*Morphology Explorer*	Nucleoli and cytoplasmic RNA	Shape
mor_MEAN_ObjectLength_Nucleoli_RNA	*Morphology Explorer*	Nucleoli and cytoplasmic RNA	Shape
mor_SD_ObjectLength_Nucleoli_RNA	*Morphology Explorer*	Nucleoli and cytoplasmic RNA	Shape
mor_MEAN_ObjectWidth_Nucleoli_RNA	*Morphology Explorer*	Nucleoli and cytoplasmic RNA	Shape
mor_SD_ObjectWidth_Nucleoli_RNA	*Morphology Explorer*	Nucleoli and cytoplasmic RNA	Shape
mor_MEAN_ObjectAngle_Nucleoli_RNA	*Morphology Explorer*	Nucleoli and cytoplasmic RNA	Shape
mor_SD_ObjectAngle_Nucleoli_RNA	*Morphology Explorer*	Nucleoli and cytoplasmic RNA	Shape
mor_MEAN_ObjectFiberLength_Nucleoli_RNA	*Morphology Explorer*	Nucleoli and cytoplasmic RNA	Shape
mor_SD_ObjectFiberLength_Nucleoli_RNA	*Morphology Explorer*	Nucleoli and cytoplasmic RNA	Shape
mor_MEAN_ObjectFiberWidth_Nucleoli_RNA	*Morphology Explorer*	Nucleoli and cytoplasmic RNA	Shape
mor_SD_ObjectFiberWidth_Nucleoli_RNA	*Morphology Explorer*	Nucleoli and cytoplasmic RNA	Shape
mor_MEAN_ObjectConvexHullAreaRatio_Nucleoli_RNA	*Morphology Explorer*	Nucleoli and cytoplasmic RNA	Area
mor_SD_ObjectConvexHullAreaRatio_Nucleoli_RNA	*Morphology Explorer*	Nucleoli and cytoplasmic RNA	Area
mor_MEAN_ObjectConvexHullPerimRatio_Nucleoli_RNA	*Morphology Explorer*	Nucleoli and cytoplasmic RNA	Shape
mor_SD_ObjectConvexHullPerimRatio_Nucleoli_RNA	*Morphology Explorer*	Nucleoli and cytoplasmic RNA	Shape

mor_MEAN_ObjectEqCircDiam_Nucleoli_RNA	*Morphology Explorer*	Nucleoli and cytoplasmic RNA	Shape
mor_SD_ObjectEqCircDiam_Nucleoli_RNA	*Morphology Explorer*	Nucleoli and cytoplasmic RNA	Shape
mor_MEAN_ObjectEqSphereVol_Nucleoli_RNA	*Morphology Explorer*	Nucleoli and cytoplasmic RNA	Shape
mor_SD_ObjectEqSphereVol_Nucleoli_RNA	*Morphology Explorer*	Nucleoli and cytoplasmic RNA	Shape
mor_MEAN_ObjectEqSphereArea_Nucleoli_RNA	*Morphology Explorer*	Nucleoli and cytoplasmic RNA	Area
mor_SD_ObjectEqSphereArea_Nucleoli_RNA	*Morphology Explorer*	Nucleoli and cytoplasmic RNA	Area
mor_MEAN_ObjectEqEllipseLWR_Nucleoli_RNA	*Morphology Explorer*	Nucleoli and cytoplasmic RNA	Shape
mor_SD_ObjectEqEllipseLWR_Nucleoli_RNA	*Morphology Explorer*	Nucleoli and cytoplasmic RNA	Shape
mor_MEAN_ObjectEqEllipseProlateVol_Nucleoli_RNA	*Morphology Explorer*	Nucleoli and cytoplasmic RNA	Shape
mor_SD_ObjectEqEllipseProlateVol_Nucleoli_RNA	*Morphology Explorer*	Nucleoli and cytoplasmic RNA	Shape
mor_MEAN_ObjectEqEllipseOblateVol_Nucleoli_RNA	*Morphology Explorer*	Nucleoli and cytoplasmic RNA	Shape
mor_SD_ObjectEqEllipseOblateVol_Nucleoli_RNA	*Morphology Explorer*	Nucleoli and cytoplasmic RNA	Shape
mor_MEAN_ObjectTotalInten_Nucleoli_RNA	*Morphology Explorer*	*Nucleoli and cytoplasmic RNA*	*Intensity*
mor_SD_ObjectTotalInten_Nucleoli_RNA	*Morphology Explorer*	*Nucleoli and cytoplasmic RNA*	*Intensity*
mor_MEAN_ObjectAvgInten_Nucleoli_RNA	*Morphology Explorer*	*Nucleoli and cytoplasmic RNA*	*Intensity*

mor_SD_ObjectAvgInten_Nucleoli_RNA	*Morphology Explorer*	*Nucleoli and cytoplasmic RNA*	*Intensity*
mor_MEAN_ObjectVarInten_Nucleoli_RNA	*Morphology Explorer*	*Nucleoli and cytoplasmic RNA*	*Intensity*
mor_SD_ObjectVarInten_Nucleoli_RNA	*Morphology Explorer*	*Nucleoli and cytoplasmic RNA*	*Intensity*
mor_MEAN_ObjectSkewInten_Nucleoli_RNA	*Morphology Explorer*	*Nucleoli and cytoplasmic RNA*	*Intensity*
mor_SD_ObjectSkewInten_Nucleoli_RNA	*Morphology Explorer*	*Nucleoli and cytoplasmic RNA*	*Intensity*
mor_MEAN_ObjectKurtInten_Nucleoli_RNA	*Morphology Explorer*	*Nucleoli and cytoplasmic RNA*	*Intensity*
mor_SD_ObjectKurtInten_Nucleoli_RNA	*Morphology Explorer*	*Nucleoli and cytoplasmic RNA*	*Intensity*
mor_MEAN_ObjectEntropyInten_Nucleoli_RNA	*Morphology Explorer*	*Nucleoli and cytoplasmic RNA*	*Intensity*
mor_SD_ObjectEntropyInten_Nucleoli_RNA	*Morphology Explorer*	*Nucleoli and cytoplasmic RNA*	*Intensity*
mor_MEAN_ObjectDiffIntenDensity_Nucleoli_RNA	*Morphology Explorer*	*Nucleoli and cytoplasmic RNA*	*Intensity*
mor_SD_ObjectDiffIntenDensity_Nucleoli_RNA	*Morphology Explorer*	*Nucleoli and cytoplasmic RNA*	*Intensity*
mor_MEAN_MemberCount_Nucleus	*Morphology Explorer*	*Nucleus*	*Count*
mor_SD_MemberCount_Nucleus	*Morphology Explorer*	*Nucleus*	*Count*
mor_MEAN_MemberOutCount_Nucleus	*Morphology Explorer*	*Nucleus*	*Count*
mor_SD_MemberOutCount_Nucleus	*Morphology Explorer*	*Nucleus*	*Count*
mor_MEAN_MemberAvgArea_Nucleus	*Morphology Explorer*	Nucleus	Area
mor_SD_MemberAvgArea_Nucleus	*Morphology Explorer*	Nucleus	Area
mor_MEAN_MemberAvgShapeP2A_Nucleus	*Morphology Explorer*	Nucleus	Shape

mor_SD_MemberAvgShapeP2A_Nucleus	*Morphology Explorer*	Nucleus	Shape
mor_MEAN_MemberAvgShapeLWR_Nucleus	*Morphology Explorer*	Nucleus	Shape
mor_SD_MemberAvgShapeLWR_Nucleus	*Morphology Explorer*	Nucleus	Shape
mor_MEAN_MemberAvgShapeBFR_Nucleus	*Morphology Explorer*	Nucleus	Shape
mor_SD_MemberAvgShapeBFR_Nucleus	*Morphology Explorer*	Nucleus	Shape
mor_MEAN_MemberAvgTotalInten_Nucleus	*Morphology Explorer*	*Nucleus*	*Intensity*
mor_SD_MemberAvgTotalInten_Nucleus	*Morphology Explorer*	*Nucleus*	*Intensity*
mor_MEAN_MemberAvgAvgInten_Nucleus	*Morphology Explorer*	*Nucleus*	*Intensity*
mor_SD_MemberAvgAvgInten_Nucleus	*Morphology Explorer*	*Nucleus*	*Intensity*
mor_MEAN_MemberAvgConvexHullAreaRatio_Nucleus	*Morphology Explorer*	Nucleus	Area
mor_SD_MemberAvgConvexHullAreaRatio_Nucleus	*Morphology Explorer*	Nucleus	Area
mor_MEAN_MemberAvgConvexHullPerimRatio_Nucleus	*Morphology Explorer*	Nucleus	Shape
mor_SD_MemberAvgConvexHullPerimRatio_Nucleus	*Morphology Explorer*	Nucleus	Shape
mor_MEAN_MemberAvgCircleDiam_Nucleus	*Morphology Explorer*	Nucleus	Shape
mor_SD_MemberAvgCircleDiam_Nucleus	*Morphology Explorer*	Nucleus	Shape
mor_MEAN_MemberAvgEllipseLWR_Nucleus	*Morphology Explorer*	Nucleus	Shape
mor_SD_MemberAvgEllipseLWR_Nucleus	*Morphology Explorer*	Nucleus	Shape
mor_MEAN_MemberObjectAreaRatio_Nucleus	*Morphology Explorer*	Nucleus	Area
mor_SD_MemberObjectAreaRatio_Nucleus	*Morphology Explorer*	Nucleus	Area
mor_MEAN_MemberObjectAreaDiff_Nucleus	*Morphology Explorer*	Nucleus	Area
mor_SD_MemberObjectAreaDiff_Nucleus	*Morphology Explorer*	Nucleus	Area
mor_MEAN_ROI_TotalInten_Nucleus	*Morphology Explorer*	*Nucleus*	*Intensity*
mor_SD_ROI_TotalInten_Nucleus	*Morphology Explorer*	*Nucleus*	*Intensity*
mor_MEAN_ROI_AvgInten_Nucleus	*Morphology Explorer*	*Nucleus*	*Intensity*
mor_SD_ROI_AvgInten_Nucleus	*Morphology Explorer*	*Nucleus*	*Intensity*

mor_MEAN_SpotFiberCount_AGP	*Morphology Explorer*	*Actin, Golgi, and plasma membrane*	*Count*
mor_SD_SpotFiberCount_AGP	*Morphology Explorer*	*Actin, Golgi, and plasma membrane*	*Count*
mor_MEAN_SpotFiberTotalArea_AGP	*Morphology Explorer*	Actin, Golgi, and plasma membrane	Area
mor_SD_SpotFiberTotalArea_AGP	*Morphology Explorer*	Actin, Golgi, and plasma membrane	Area
mor_MEAN_SpotFiberAvgArea_AGP	*Morphology Explorer*	Actin, Golgi, and plasma membrane	Area
mor_SD_SpotFiberAvgArea_AGP	*Morphology Explorer*	Actin, Golgi, and plasma membrane	Area
mor_MEAN_FiberAlign1_AGP	*Morphology Explorer*	Actin, Golgi, and plasma membrane	Shape
mor_SD_FiberAlign1_AGP	*Morphology Explorer*	Actin, Golgi, and plasma membrane	Shape
mor_MEAN_ROI_FiberAlign2_AGP	*Morphology Explorer*	Actin, Golgi, and plasma membrane	Shape
mor_SD_ROI_FiberAlign2_AGP	*Morphology Explorer*	Actin, Golgi, and plasma membrane	Shape
mor_MEAN_ROI_TotalInten_AGP	*Morphology Explorer*	*Actin, Golgi, and plasma membrane*	*Intensity*
mor_SD_ROI_TotalInten_AGP	*Morphology Explorer*	*Actin, Golgi, and plasma membrane*	*Intensity*
mor_MEAN_ROI_AvgInten_AGP	*Morphology Explorer*	*Actin, Golgi, and plasma membrane*	*Intensity*
mor_SD_ROI_AvgInten_AGP	*Morphology Explorer*	*Actin, Golgi, and plasma membrane*	*Intensity*
mor_MEAN_ROI_VarInten_AGP	*Morphology Explorer*	*Actin, Golgi, and plasma membrane*	*Intensity*

mor_SD_ROI_VarInten_AGP	*Morphology Explorer*	*Actin, Golgi, and plasma membrane*	*Intensity*
mor_MEAN_ROI_SkewInten_AGP	*Morphology Explorer*	*Actin, Golgi, and plasma membrane*	*Intensity*
mor_SD_ROI_SkewInten_AGP	*Morphology Explorer*	*Actin, Golgi, and plasma membrane*	*Intensity*
mor_MEAN_ROI_KurtInten_AGP	*Morphology Explorer*	*Actin, Golgi, and plasma membrane*	*Intensity*
mor_SD_ROI_KurtInten_AGP	*Morphology Explorer*	*Actin, Golgi, and plasma membrane*	*Intensity*
mor_MEAN_ROI_EntropyInten_AGP	*Morphology Explorer*	*Actin, Golgi, and plasma membrane*	*Intensity*
mor_SD_ROI_EntropyInten_AGP	*Morphology Explorer*	*Actin, Golgi, and plasma membrane*	*Intensity*
mor_MEAN_ROI_DiffIntenDensity_AGP	*Morphology Explorer*	*Actin, Golgi, and plasma membrane*	*Intensity*
mor_SD_ROI_DiffIntenDensity_AGP	*Morphology Explorer*	*Actin, Golgi, and plasma membrane*	*Intensity*
mor_MEAN_ROI_MaxCoocInten_AGP	*Morphology Explorer*	*Actin, Golgi, and plasma membrane*	*Intensity*
mor_SD_ROI_MaxCoocInten_AGP	*Morphology Explorer*	*Actin, Golgi, and plasma membrane*	*Intensity*
mor_MEAN_ROI_ContrastCoocInten_AGP	*Morphology Explorer*	*Actin, Golgi, and plasma membrane*	*Intensity*
mor_SD_ROI_ContrastCoocInten_AGP	*Morphology Explorer*	*Actin, Golgi, and plasma membrane*	*Intensity*
mor_MEAN_ROI_EntropyCoocInten_AGP	*Morphology Explorer*	*Actin, Golgi, and plasma membrane*	*Intensity*
mor_SD_ROI_EntropyCoocInten_AGP	*Morphology Explorer*	*Actin, Golgi, and plasma membrane*	*Intensity*

mor_MEAN_ROI_ASMCoocInten_AGP	*Morphology Explorer*	*Actin, Golgi, and plasma membrane*	*Intensity*
mor_SD_ROI_ASMCoocInten_AGP	*Morphology Explorer*	*Actin, Golgi, and plasma membrane*	*Intensity*
mor_MEAN_SpotFiberCount_ER	*Morphology Explorer*	*Endoplasmic reticulum*	*Count*
mor_SD_SpotFiberCount_ER	*Morphology Explorer*	*Endoplasmic reticulum*	*Count*
mor_MEAN_SpotFiberTotalArea_ER	*Morphology Explorer*	Endoplasmic reticulum	Area
mor_SD_SpotFiberTotalArea_ER	*Morphology Explorer*	Endoplasmic reticulum	Area
mor_MEAN_SpotFiberAvgArea_ER	*Morphology Explorer*	Endoplasmic reticulum	Area
mor_SD_SpotFiberAvgArea_ER	*Morphology Explorer*	Endoplasmic reticulum	Area
mor_MEAN_FiberAlign1_ER	*Morphology Explorer*	Endoplasmic reticulum	Shape
mor_SD_FiberAlign1_ER	*Morphology Explorer*	Endoplasmic reticulum	Shape
mor_MEAN_ROI_FiberAlign2_ER	*Morphology Explorer*	Endoplasmic reticulum	Shape
mor_SD_ROI_FiberAlign2_ER	*Morphology Explorer*	Endoplasmic reticulum	Shape
mor_MEAN_ROI_TotalInten_ER	*Morphology Explorer*	*Endoplasmic reticulum*	*Intensity*
mor_SD_ROI_TotalInten_ER	*Morphology Explorer*	*Endoplasmic reticulum*	*Intensity*
mor_MEAN_ROI_AvgInten_ER	*Morphology Explorer*	*Endoplasmic reticulum*	*Intensity*
mor_SD_ROI_AvgInten_ER	*Morphology Explorer*	*Endoplasmic reticulum*	*Intensity*
mor_MEAN_ROI_VarInten_ER	*Morphology Explorer*	*Endoplasmic reticulum*	*Intensity*
mor_SD_ROI_VarInten_ER	*Morphology Explorer*	*Endoplasmic reticulum*	*Intensity*
mor_MEAN_ROI_SkewInten_ER	*Morphology Explorer*	*Endoplasmic reticulum*	*Intensity*
mor_SD_ROI_SkewInten_ER	*Morphology Explorer*	*Endoplasmic reticulum*	*Intensity*
mor_MEAN_ROI_KurtInten_ER	*Morphology Explorer*	*Endoplasmic reticulum*	*Intensity*
mor_SD_ROI_KurtInten_ER	*Morphology Explorer*	*Endoplasmic reticulum*	*Intensity*
mor_MEAN_ROI_EntropyInten_ER	*Morphology Explorer*	*Endoplasmic reticulum*	*Intensity*
mor_SD_ROI_EntropyInten_ER	*Morphology Explorer*	*Endoplasmic reticulum*	*Intensity*

mor_MEAN_ROI_DiffIntenDensity_ER	*Morphology Explorer*	*Endoplasmic reticulum*	*Intensity*
mor_SD_ROI_DiffIntenDensity_ER	*Morphology Explorer*	*Endoplasmic reticulum*	*Intensity*
mor_MEAN_ROI_MaxCoocInten_ER	*Morphology Explorer*	*Endoplasmic reticulum*	*Intensity*
mor_SD_ROI_MaxCoocInten_ER	*Morphology Explorer*	*Endoplasmic reticulum*	*Intensity*
mor_MEAN_ROI_ContrastCoocInten_ER	*Morphology Explorer*	*Endoplasmic reticulum*	*Intensity*
mor_SD_ROI_ContrastCoocInten_ER	*Morphology Explorer*	*Endoplasmic reticulum*	*Intensity*
mor_MEAN_ROI_EntropyCoocInten_ER	*Morphology Explorer*	*Endoplasmic reticulum*	*Intensity*
mor_SD_ROI_EntropyCoocInten_ER	*Morphology Explorer*	*Endoplasmic reticulum*	*Intensity*
mor_MEAN_ROI_ASMCoocInten_ER	*Morphology Explorer*	*Endoplasmic reticulum*	*Intensity*
mor_SD_ROI_ASMCoocInten_ER	*Morphology Explorer*	*Endoplasmic reticulum*	*Intensity*
mor_MEAN_SpotFiberCount_Mito	*Morphology Explorer*	*Mitochondria*	*Count*
mor_SD_SpotFiberCount_Mito	*Morphology Explorer*	*Mitochondria*	*Count*
mor_MEAN_SpotFiberTotalArea_Mito	*Morphology Explorer*	Mitochondria	Area
mor_SD_SpotFiberTotalArea_Mito	*Morphology Explorer*	Mitochondria	Area
mor_MEAN_SpotFiberAvgArea_Mito	*Morphology Explorer*	Mitochondria	Area
mor_SD_SpotFiberAvgArea_Mito	*Morphology Explorer*	Mitochondria	Area
mor_MEAN_FiberAlign1_Mito	*Morphology Explorer*	Mitochondria	Shape
mor_SD_FiberAlign1_Mito	*Morphology Explorer*	Mitochondria	Shape
mor_MEAN_ROI_FiberAlign2_Mito	*Morphology Explorer*	Mitochondria	Shape
mor_SD_ROI_FiberAlign2_Mito	*Morphology Explorer*	Mitochondria	Shape
mor_MEAN_ROI_TotalInten_Mito	*Morphology Explorer*	*Mitochondria*	*Intensity*
mor_SD_ROI_TotalInten_Mito	*Morphology Explorer*	*Mitochondria*	*Intensity*
mor_MEAN_ROI_AvgInten_Mito	*Morphology Explorer*	*Mitochondria*	*Intensity*
mor_SD_ROI_AvgInten_Mito	*Morphology Explorer*	*Mitochondria*	*Intensity*
mor_MEAN_ROI_VarInten_Mito	*Morphology Explorer*	*Mitochondria*	*Intensity*

mor_SD_ROI_VarInten_Mito	*Morphology Explorer*	*Mitochondria*	*Intensity*
mor_MEAN_ROI_SkewInten_Mito	*Morphology Explorer*	*Mitochondria*	*Intensity*
mor_SD_ROI_SkewInten_Mito	*Morphology Explorer*	*Mitochondria*	*Intensity*
mor_MEAN_ROI_KurtInten_Mito	*Morphology Explorer*	*Mitochondria*	*Intensity*
mor_SD_ROI_KurtInten_Mito	*Morphology Explorer*	*Mitochondria*	*Intensity*
mor_MEAN_ROI_EntropyInten_Mito	*Morphology Explorer*	*Mitochondria*	*Intensity*
mor_SD_ROI_EntropyInten_Mito	*Morphology Explorer*	*Mitochondria*	*Intensity*
mor_MEAN_ROI_DiffIntenDensity_Mito	*Morphology Explorer*	*Mitochondria*	*Intensity*
mor_SD_ROI_DiffIntenDensity_Mito	*Morphology Explorer*	*Mitochondria*	*Intensity*
mor_MEAN_ROI_MaxCoocInten_Mito	*Morphology Explorer*	*Mitochondria*	*Intensity*
mor_SD_ROI_MaxCoocInten_Mito	*Morphology Explorer*	*Mitochondria*	*Intensity*
mor_MEAN_ROI_ContrastCoocInten_Mito	*Morphology Explorer*	*Mitochondria*	*Intensity*
mor_SD_ROI_ContrastCoocInten_Mito	*Morphology Explorer*	*Mitochondria*	*Intensity*
mor_MEAN_ROI_EntropyCoocInten_Mito	*Morphology Explorer*	*Mitochondria*	*Intensity*
mor_SD_ROI_EntropyCoocInten_Mito	*Morphology Explorer*	*Mitochondria*	*Intensity*
mor_MEAN_ROI_ASMCoocInten_Mito	*Morphology Explorer*	*Mitochondria*	*Intensity*
mor_SD_ROI_ASMCoocInten_Mito	*Morphology Explorer*	*Mitochondria*	*Intensity*

Features in italics exhibited strong correlation with fluorescence intensity and were filtered out to avoid bias from sample-dependent dye quenching effects. These features were not included in the final morphological profiling analysis.

Table S4. Cluster assignments of IHSS samples based on hierarchical clustering analysis (HCA).

Cluster Assignment	Sample	IHSS Cat.No.	Sources	Fractions
Cluster1	Elliott Soil Humic Acid Standard I	1S102H	Elliott Soil	Humic Acid
	Elliott Soil Humic Acid Standard IV	4S102H	Elliott Soil	Humic Acid
	Elliott Soil Humic Acid Standard V	5S102H	Elliott Soil	Humic Acid
	Elliott Soil Humic Acid Standard VI	6S102H	Elliott Soil	Humic Acid
	Elliott Soil Humic Acid Reference I	1R102H	Elliott Soil	Humic Acid
	Leonardite Humic Acid Standard I	1S104H	Leonardite	Humic Acid
	Pahokee Peat Humic Acid Standard I	1S103H	Pahokee Peat	Humic Acid
	Summit Hill Soil Humic Acid Reference I	1R106H	Summit Hill Soil	Humic Acid
	Pahokee Peat Humic Acid Reference I	1R103H	Pahokee Peat	Humic Acid
Cluster2	Pony Lake Fulvic Acid Reference I	1R109F	Pony Lake	Fulvic Acid
Cluster3	Elliott Soil Fulvic Acid Standard I	1S102F	Elliott Soil	Fulvic Acid
	Elliott Soil Fulvic Acid Standard II	2S102F	Elliott Soil	Fulvic Acid
	Elliott Soil Fulvic Acid Standard V	5S102F	Elliott Soil	Fulvic Acid
	Elliott Soil Fulvic Acid Standard VI	6S102F	Elliott Soil	Fulvic Acid
	Pahokee Peat Fulvic Acid Standard I	1S103F	Pahokee Peat	Fulvic Acid
	Pahokee Peat Fulvic Acid Standard II	2S103F	Pahokee Peat	Fulvic Acid
	Pahokee Peat Fulvic Acid Reference I	1R103F	Pahokee Peat	Fulvic Acid
Cluster4	Suwannee River Humic Acid Standard I	1S101H	Suwannee River	Humic Acid
	Suwannee River Humic Acid Standard II	2S101H	Suwannee River	Humic Acid
	Suwannee River Humic Acid Standard III	3S101H	Suwannee River	Humic Acid
	Suwannee River Humic Acid Reference I	1R101H	Suwannee River	Humic Acid

	Waskish Peat Humic Acid Reference I	1R107H	Waskish Peat	Humic Acid
	Nordic Aquatic Humic Acid Reference I	1R105H	Nordic Reservoir	Humic Acid
	Suwannee River Fulvic Acid Standard I	1S101F	Suwannee River	Fulvic Acid
	Suwannee River Fulvic Acid Standard II	2S101F	Suwannee River	Fulvic Acid
	Suwannee River Fulvic Acid Standard III	3S101F	Suwannee River	Fulvic Acid
	Waskish Peat Fulvic Acid Reference I	1R107F	Waskish Peat	Fulvic Acid
	Suwannee River Fulvic Acid Reference I	1R101F	Suwannee River	Fulvic Acid
	Nordic Aquatic Fulvic Acid Reference I	1R105F	Nordic Reservoir	Fulvic Acid
	Suwannee River Natural Organic Matter I	1R101N	Suwannee River	Natural Organic Matter
	Suwannee River Natural Organic Matter II	2R101N	Suwannee River	Natural Organic Matter
	Nordic Reservoir Natural Organic Matter I	1R108N	Nordic Reservoir	Natural Organic Matter
	Upper Mississipi River Natural Organic Matter I	1R110N	Mississippi River	Natural Organic Matter

Table S5. List of mass differences and corresponding elemental transformations used for network analysis. Each transformation is described by its exact mass difference, formula difference, and chemical interpretation.

Mass (Da)	Transformation	Type
2.01565	+H2	Hydrogenation / Dehydrogenation
25.97927	-H2+CO	Carbonylation / Dehydrogenation
12.00000	+C	Carbon Addition / Condensation
14.01565	+CH2	Alkylation
1.97927	-CH2+O	Oxidative Cleavage
15.99492	+O	Oxygenation / Hydroxylation / carbonylation
27.99492	+CO	Carbonylation
26.01565	+C2H2	Aromatic Condensation
28.03130	+C2H4	Alkylation
30.01057	+CH2O	Hydroxymethylation / Formylation
31.98983	+O2	Oxidation
42.01057	+C2H2O	Acylation
43.98983	+CO2	Carboxylation
44.02622	+C2H4O	O-alkylation
26.05204	-O+C3H6	Deoxygenative Alkylation
55.98983	+C2O2	Dicarbonylation
56.02622	+C3H4O	Acylation
70.00548	+C3H2O2	Dicarbonylation
70.04187	+C4H6O	Unsaturated Acylation
84.02113	+C4H4O2	Acylation
0.98402	-NH+O	Oxidative Deamination
1.03163	-O+NH3	Deoxygenative Amination

15.0109	+NH	Amination / Deamination
15.02348	+CH3	Methylation / Demethylation
18.01057	+H2O	Hydration / Hydroxylation
42.0218	+CH2N2	Diazomethylation
43.00581	+CHNO	Aminocarbonylation
43.0422	+C2H5N	Aminoalkylation
57.02146	+C2H3NO	Aminocarbonylation
57.05785	+C3H7N	Aminoalkylation
59.03711	+C2H5NO	Aminoacylation
60.02113	+C2H4O2	Acylation
69.02146	+C3H3ON	Aminocarbonylation
70.0531	+C3H6N2	Diamination
71.03711	+C3H5ON	Aminocarbonylation
71.98475	+C2O3	Dicarbonylation
72.05752	+C4H8O	O-alkylation
73.01638	+C2H3NO2	Aminocarboxylation
73.05276	+C3H7NO	Aminoacylation
74.03678	+C3H6O2	Acylation

References

(1) Tziotis, D.; Hertkorn, N.; Schmitt-Kopplin, P. Kendrick-analogous network visualisation of ion cyclotron resonance Fourier transform mass spectra: improved options for the assignment of elemental compositions and the classification of organic molecular complexity. European Journal of Mass Spectrometry **2011**, 17 (4), 415-421.

(2) Catalán, N.; Rofner, C.; Verpoorter, C.; Pérez, M. T.; Dittmar, T.; Tranvik, L.; Sommaruga, R.; Peter, H. Treeline displacement may affect lake dissolved organic matter processing at high latitudes and altitudes. Nature communications **2024**, 15 (1), 2640.

(3) Koch, B. P.; Dittmar, T. From mass to structure: An aromaticity index for high-resolution mass data of natural organic matter. Rapid communications in mass spectrometry **2006**, 20 (5), 926-932.

(4) Glöckler, D.; Harir, M.; Schmitt-Kopplin, P.; Elsner, M.; Bakkour, R. Selectivity of β-cyclodextrin polymer toward aquatic contaminants: insights from ultrahigh-resolution mass spectrometry of dissolved organic matter. Analytical chemistry **2023**, 95 (42), 15505-15513.

(5) Kim, S.; Kramer, R. W.; Hatcher, P. G. Graphical method for analysis of ultrahigh-resolution broadband mass spectra of natural organic matter, the van Krevelen diagram. Analytical chemistry **2003**, 75 (20), 5336-5344.

(6) Gaspar, A.; Harir, M.; Lucio, M.; Hertkorn, N.; Schmitt-Kopplin, P. Targeted borate complex formation as followed with electrospray ionization Fourier transform ion cyclotron mass spectrometry: monomolecular model system and polyborate formation. Rapid Communications in Mass Spectrometry: An International Journal Devoted to the Rapid Dissemination of Up-to-the-Minute Research in Mass Spectrometry **2008**, 22 (20), 3119-3129.
